# Functional and quality of life outcomes of localised prostate cancer treatments (Prostate Testing for Cancer and Treatment [ProtecT] study)

**DOI:** 10.1111/bju.15739

**Published:** 2022-05-03

**Authors:** Janet Athene Lane, Jenny L. Donovan, Grace J. Young, Michael Davis, Eleanor I. Walsh, Kerry N.L. Avery, Jane M. Blazeby, Malcolm D. Mason, Richard M. Martin, Tim J. Peters, Emma L. Turner, Julia Wade, Prasad Bollina, James W.F. Catto, Alan Doherty, David Gillatt, Vincent Gnanapragasam, Owen Hughes, Roger Kockelbergh, Howard Kynaston, Jon Oxley, Alan Paul, Edgar Paez, Derek J. Rosario, Edward Rowe, John Staffurth, David E. Neal, Freddie C. Hamdy, Chris Metcalfe, for the Prostate Testing for Cancer and Treatment (ProtecT) Study Group, Andrew Doble, Philip Powell, Stephen Prescott, John B. Anderson, Jonathan Aning, Garett Durkan, Anthony Kouparis, Hing Leung, Param Mariappan, Alan McNeill, Raj Persad, Hartwig Schwaibold, David Tulloch, Michael Wallace, Susan Bonnington, Lynne Bradshaw, Deborah Cooper, Emma Elliott, Phillipa Herbert, Peter Holding, Joanne Howson, Amanda Jones, Teresa Lennon, Norma Lyons, Hilary Moody, Claire Plumb, Tricia O'Sullivan, Elizabeth Salter, Pauline Thompson, Sarah Tidball, Jan Blaikie, Catherine Grey, Tonia Adam, Sarah Askew, Sharon Atkinson, Tim Baynes, Carole Brain, Viv Breen, Sarah Brunt, Sean Bryne, Jo Bythem, Jenny Clarke, Jenny Cloete, Susan Dark, Gill Davis, Rachael De La Rue, Jane Denizot, Elspeth Dewhurst, Anna Dimes, Nicola Dixon, Penny Ebbs, Ingrid Emmerson, Jill Ferguson, Ali Gadd, Lisa Geoghegan, Alison Grant, Collette Grant, Rosemary Godfrey, Louise Goodwin, Susie Hall, Liz Hart, Andrew Harvey, Chloe Hoult, Sarah Hawkins, Sharon Holling, Alastair Innes, Sue Kilner, Fiona Marshall, Louise Mellen, Andrea Moore, Sally Napier, Julie Needham, Kevin Pearse, Anna Pisa, Mark Rees, Ellie Richards, Lindsay Robson, Janet Roxburgh, Nikki Samuel, Irene Sharkey, Michael Slater, Donna Smith, Pippa Taggart, Helen Taylor, Vicky Taylor, Ayesha Thomas, Briony Tomkies, Nicola Trewick, Claire Ward, Christy Walker, Ayesha Williams, Colin Woodhouse, Elizabeth Wyber, Amit Bahl, Richard Benson, Mark Beresford, Catherine Ferguson, John Graham, Chris Herbert, Grahame Howard, Nick James, Peter Kirkbride, Alastair Law, Carmel Loughrey, Duncan McClaren, Helen Patterson, Ian Pedley, Trevor Roberts, Angus Robinson, Simon Russell, Paul Symonds, Narottam Thanvi, Subramaniam Vasanthan, Paula Wilson, Mary Robinson, Selina Bhattarai, Neeta Deshmukh, John Dormer, Malee Fernando, John Goepel, David Griffiths, Ken Grigor, Nick Mayer, Murali Varma, Anne Warren, Helen Appleby, Dominic Ash, Dean Aston, Steven Bolton, Graham Chalmers, John Conway, Nick Early, Tony Geater, Lynda Goddall, Claire Heymann, Deborah Hicks, Liza Jones, Susan Lamb, Geoff Lambert, Gill Lawrence, Geraint Lewis, John Lilley, Aileen MacLeod, Pauline Massey, Alison McQueen, Rollo Moore, Lynda Penketh, Janet Potterton, Neil Roberts, Helen Showler, Pam Shuttleworth, Stephen Slade, Alasdair Steele, James Swinscoe, Marie Tiffany, John Townley, Jo Treeby, Michael Weston, Joyce Wilkinson, Lorraine Williams, Lucy Wills, Owain Woodley, Sue Yarrow, Lucy Brindle, Linda Davies, Dan Dedman, Elizabeth Down, Hanan Khazragui, Sian Noble, Hilary Taylor, Marta Tazewell, Susan Baker, Elizabeth Bellis‐Sheldon, Chantal Bougard, Joanne Bowtell, Catherine Brewer, Chris Burton, Jennie Charlton, Nicholas Christoforou, Rebecca Clark, Susan Coull, Christine Croker, Rosemary Currer, Claire Daisey, Gill Delaney, Rose Donohue, Jane Drew, Rebecca Farmer, Susan Fry, Jean Haddow, Alex Hale, Susan Halpin, Belle Harris, Barbara Hattrick, Sharon Holmes, Helen Hunt, Vicky Jackson, Donna Johnson, Mandy Le Butt, Jo Leworthy, Tanya Liddiatt, Alex Martin, Jainee Mauree, Susan Moore, Gill Moulam, Jackie Mutch, Kathleen Parker, Christopher Pawsey, Michelle Purdie, Teresa Robson, Lynne Smith, Carole Stenton, Tom Steuart‐Feilding, Beth Stott, Chris Sully, Caroline Sutton, Carol Torrington, Zoe Wilkins, Sharon Williams, Andrea Wilson, Ashleigh Weaver, Peter Albertsen, Jan Adolfsson, Michael Baum, Anthony Koupparis, Jon McFarlane, Colette Reid, Mary Robinson, Anthony Zietman, Elizabeth Hill, Siaw Yein Ng, Naomi Williams, Jessica Toole, Charlotte Davies, Laura Hughes, Mari‐Anne Rowlands, Lindsey Bell, Sean Harrison, Adrian Grant, Ian Roberts, Deborah Ashby, Richard Cowan, Peter Fayers, Killian Mellon, James N'Dow, Tim O'Brien, Michael Sokhal, David Dearnaley, Fritz Schröder, Tracy Roberts

**Affiliations:** ^1^ Bristol Medical School University of Bristol Bristol UK; ^2^ Bristol Medical School Bristol Trials Centre University of Bristol Bristol UK; ^3^ Bristol Medical School NIHR Bristol Biomedical Research Centre University of Bristol Bristol UK; ^4^ School of Medicine Cardiff University Cardiff UK; ^5^ Department of Urology and Surgery Western General Hospital University of Edinburgh Edinburgh UK; ^6^ Academic Urology Unit University of Sheffield Sheffield UK; ^7^ Department of Urology Queen Elizabeth Hospital Birmingham UK; ^8^ Department of Urology Southmead Hospital and Bristol Urological Institute Bristol UK; ^9^ Department of Urology Addenbrooke’s Hospital Cambridge UK; ^10^ Department of Urology Cardiff and Vale University Health Board Cardiff UK; ^11^ Department of Urology University Hospitals of Leicester Leicester UK; ^12^ Department of Cellular Pathology North Bristol NHS Trust Bristol UK; ^13^ Department of Urology Leeds Teaching Hospitals NHS Trust Leeds UK; ^14^ Department of Urology Freeman Hospital Newcastle upon Tyne UK; ^15^ Division of Cancer and Genetics School of Medicine Cardiff University Cardiff UK; ^16^ Nuffield Department of Surgical Sciences University of Oxford Oxford UK; ^17^ NIHR Oxford Biomedical Research Centre Oxford University Hospitals NHS Foundation Trust and University of Oxford UK

**Keywords:** localised prostate cancer, treatments, patient‐reported outcomes, functional outcomes, quality of life, #PCSM, #ProstateCancer, #uroonc

## Abstract

**Objective:**

To investigate the functional and quality of life (QoL) outcomes of treatments for localised prostate cancer and inform treatment decision‐making.

**Patients and Methods:**

Men aged 50–69 years diagnosed with localised prostate cancer by prostate‐specific antigen testing and biopsies at nine UK centres in the Prostate Testing for Cancer and Treatment (ProtecT) trial were randomised to, or chose one of, three treatments. Of 2565 participants, 1135 men received active monitoring (AM), 750 a radical prostatectomy (RP), 603 external‐beam radiotherapy (EBRT) with concurrent androgen‐deprivation therapy (ADT) and 77 low‐dose‐rate brachytherapy (BT, not a randomised treatment). Patient‐reported outcome measures (PROMs) completed annually for 6 years were analysed by initial treatment and censored for subsequent treatments. Mixed effects models were adjusted for baseline characteristics using propensity scores.

**Results:**

Treatment‐received analyses revealed different impacts of treatments over 6 years. Men remaining on AM experienced gradual declines in sexual and urinary function with age (e.g., increases in erectile dysfunction from 35% of men at baseline to 53% at 6 years and nocturia similarly from 20% to 38%). Radical treatment impacts were immediate and continued over 6 years. After RP, 95% of men reported erectile dysfunction persisting for 85% at 6 years, and after EBRT this was reported by 69% and 74%, respectively (*P* < 0.001 compared with AM). After RP, 36% of men reported urinary leakage requiring at least 1 pad/day, persisting for 20% at 6 years, compared with no change in men receiving EBRT or AM (*P* < 0.001). Worse bowel function and bother (e.g., bloody stools 6% at 6 years and faecal incontinence 10%) was experienced by men after EBRT than after RP or AM (*P* < 0.001) with lesser effects after BT. No treatment affected mental or physical QoL.

**Conclusion:**

Treatment decision‐making for localised prostate cancer can be informed by these 6‐year functional and QoL outcomes.

## Introduction

Radical treatments for localised prostate cancer have shown oncological benefits in several randomised trials compared to active surveillance or watchful waiting with comparable disease‐specific survival [1]. To assess the ‘trade‐off’ between oncological outcomes and future quality of life (QoL) after treatment, informed shared treatment decisions require knowledge of the sexual, urinary and bowel functional and QoL impacts of localised prostate cancer treatments, which are optimally assessed with patient‐reported outcome measures (PROMs). Several cohorts [2, 3] and the UK National Institute of Health Research (NIHR) Prostate Testing for Cancer and Treatment (ProtecT) randomised trial [4, 5], which diagnosed 1643 men with population‐based PSA testing and biopsies [5] have published PROMs, but differences between studies hinder their utility for clinicians and patients [6]. For example, PROMs from the Comparative Effectiveness Analysis of Surgery and Radiation (CEASAR) cohort [2] and the Prostate Cancer Comparative Effectiveness and Survivorship Study (ProCESS) [3] were analysed according to treatments received, whilst ProtecT analysed by intention‐to‐treat (ITT) i.e., random allocation. The ProtecT ITT analysis potentially underestimates the harms of radical treatments, as 54% of men randomised to active monitoring (AM) changed treatment over 10 years’ follow‐up [5]. Studies also differ in the active surveillance comparator for radical treatment impacts, PROMs, follow‐up periods and response rates, which complicates the interpretation of findings for patients and clinicians. The United States Preventive Services Task Force systematic review of prostate cancer [6] could not meta‐analyse radiotherapy (RT) PROMs due to their heterogeneity across studies.

The objective of the present study was to analyse the functional and QoL PROMs for treatments received by men randomised to or selecting their own treatments in the ProtecT trial over 6 years, to generate long‐term side‐effect profiles and assist patients and clinicians in treatment decision‐making. We also compared these PROMs with those experienced by men who received radical treatments after a period of AM and by age group.

## Patients and Methods

### Study Design and Participants

Men aged 50–69 years were invited for PSA testing at primary care practices at nine UK urology centres between 1999 and 2009 [7]. Of 2640 men diagnosed with clinically localised prostate cancer following biopsies, 1643 were randomised to AM or three‐dimensional‐conformal external‐beam RT (EBRT; a precursor to intensity‐modulated RT [IMRT], without image‐guided RT, 74 Gy in 37 fractions to the prostate) with 3–6 months neoadjuvant and androgen‐deprivation therapy (ADT) concurrent with RT, or open retropubic radical prostatectomy (RP) [8]. The AM protocol included PSA testing every 6–12 months (3‐monthly in the first year) with an increase of ≥50% over a 12‐month period triggering a clinical review (including potentially imaging and/or repeat biopsies as required) about possible change of management. There were 997 men who declined randomisation and chose a ProtecT treatment or low‐dose‐rate brachytherapy (BT) [9]. Research nurse follow‐up, with supervision by a senior urologist, was by annual record review and a participant visit. Ethics committee approval was obtained from the UK Trent Multicentre Research Ethics Committee (01/4/025). Participants provided written informed consent. At baseline, participants reported their age and ethnicity, and nurses collected other sociodemographic and clinical information.

### Outcomes

Validated PROMs were collected until the median 10‐year analysis timepoint (23 November 2015) at diagnostic biopsy clinics, 6 months, then annually from the time of randomisation or treatment choice by mailed questionnaires with a structured reminder system [8]. Functional impacts were evaluated with the International Consultation on Incontinence Questionnaire (ICIQ) [10], the ICS urinary ICS*male*SF [11] and the 50‐item Expanded Prostate Index Composite (EPIC‐50, added in 2005 before EPIC‐26 was available, excluding the hormone domain to reduce participant burden as related only to EBRT at diagnosis) [12]. The Hospital Anxiety and Depression Scale (HADS) [13], Medical Outcomes Study 12‐Item Short Form Health Survey (SF‐12) [14] measured general health‐related QoL. Key items and QoL scores were presented graphically as previously [4]: urinary leakage (EPIC – at least 1 incontinence pad/day); erectile dysfunction (EPIC – erections not firm enough for intercourse); bloody stools (EPIC – ≥50% of the time); nocturia (ICS*male*SF – urinating at least once a night); anxiety and depression (HADS case score of ≥8); general mental and physical function (SF‐12 subscores). EPIC scores and SF‐12 subscales were reversed for display (higher score – worse impact) to align with other outcomes.

### Statistical Analysis

This analysis of treatments in the randomised and ‘treatment choice’ cohorts (*n* = 2565) (as in the trial statistical analysis plan) [15] included men whose treatment commenced within 12 months of diagnosis. As previously [4, 16, 17], treatment was considered received for AM if there were at least two PSA tests recorded within 12 months of diagnosis and EBRT (including ADT) or BT was completed within 15 months. The date radical treatments commenced was time zero (randomisation/choice date for AM) and time on treatment calculated thereafter. Questionnaire data were censored on subsequent prostate cancer treatments to identify specific impacts of initial treatments.

Initially, four‐way comparisons of items by treatments were conducted, using a likelihood‐ratio test to compare a multilevel mixed effects linear regression model that included treatment as a covariate, to one where it was excluded. Two‐level models were used to incorporate the repeated PROMs for each individual over the 6 years following primary treatment. The mixed model appropriately distinguishes the within participant (level 1, the repeated measures) and between participant (level 2) variation, incorporating the latter as a normally distributed random effect. The randomised and treatment choice cohorts were previously shown to be comparable at baseline [9] and so were combined as previously for the clinical outcomes [17]. All analyses were adjusted for cohort (randomised or choice). Baseline PROMs were excluded from models as they can bias comparison of non‐randomised groups [18]. PROMs are presented as line graphs with each point including all questionnaires completed within 12 months (e.g., point 2 captures questionnaires completed between 1 and 2 years after treatment date and referred to as 2 years in the text and 1–2 years in tables). Men who had a radical treatment initially were compared with those who changed to the same radical treatment after AM on a treatment‐received basis with responses censored at a third treatment (time zero at second treatment date).

As symptoms were influenced by age at baseline [8], we also compared primary treatments for key items and scores by younger (<65 years) and older age (≥65 years) groups (BT excluded due to low numbers). The likelihood‐ratio tests compared models with and without an interaction term between treatment received and age (continuous) in years. All analyses used STATA 16.1 (StataCorp, College Station, TX, USA).

## Results

Of 2565 men diagnosed with localised prostate cancer, 1135 received AM (628 randomised, 507 treatment choice) and over 6 years 174 of those men subsequently underwent surgery (15%) or 144 EBRT (13%) (Fig. S1). In total, 470 men received protocol EBRT, and 441 with ADT (93.8%), eight others did not receive ADT and 21 had missing ADT data. Non‐protocol EBRT was received by 34 men (mainly in the feasibility phase) with 13 with ADT (eight missing data) and there were 14 men who ceased EBRT due to complications (85 with missing dose data). Of 750 open RPs (488 randomised, 262 treatment choice), 427 men had a bilateral nerve‐sparing procedure and 60 a unilateral procedure, 119 men had non‐nerve‐sparing surgery and in 44 men nerve sparing was not recorded (Fig. S1). Questionnaire response rates exceeded 75% over 6 years (exemplars shown in Table S1).

### Baseline Characteristics and Function

**Fig. 1:**
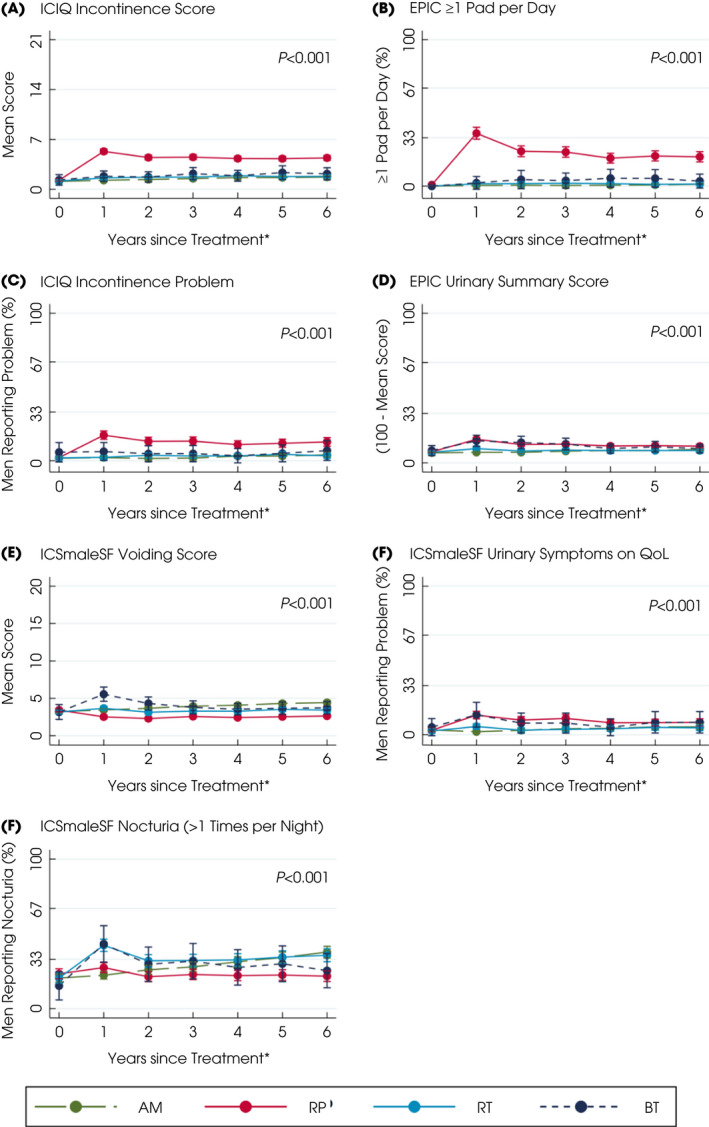
Patient‐reported urinary symptoms and QoL after primary treatments for localised prostate cancer over 6 years. *Questionnaires completed for e.g., year 2 as between 1 and 2 years after treatment. Points are estimated means from models with error bars representing 95% CIs. Higher scores or percentages indicate worse symptoms. *P* value based on likelihood‐ratio test for overall comparison of treatments. [Colour figure can be viewed at wileyonlinelibrary.com]

**Fig. 2:**
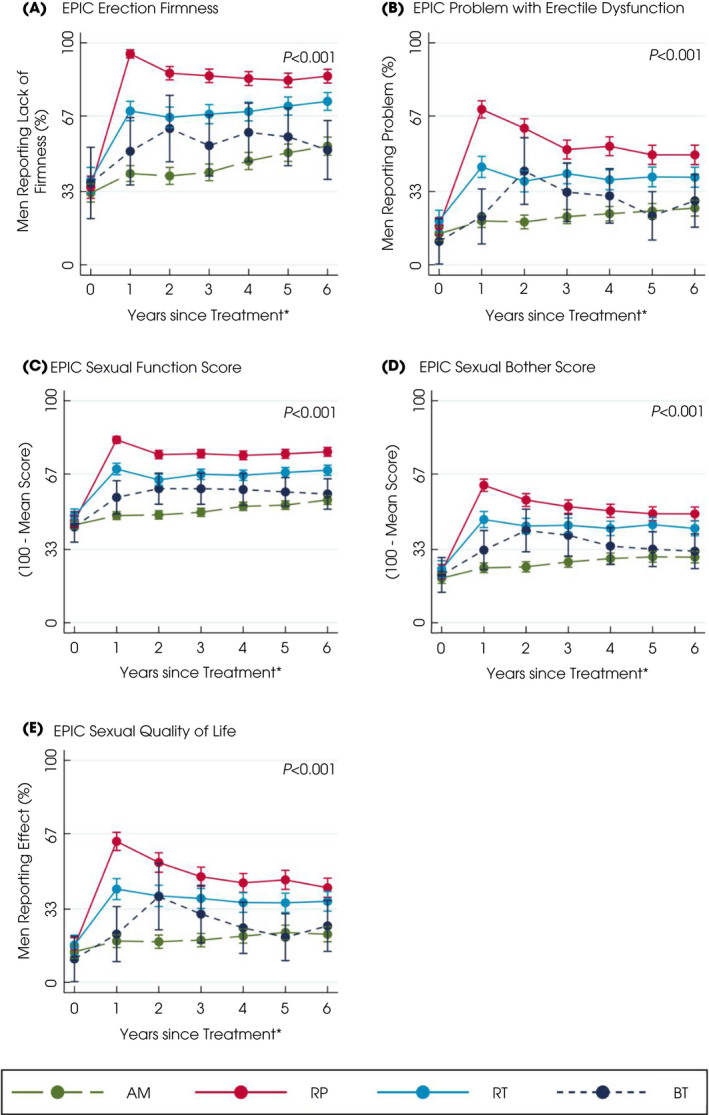
Patient‐reported sexual symptoms and QoL after primary treatments for localised prostate cancer over 6 years. *Questionnaires completed for e.g., year 2 as between 1 and 2 years after treatment. Higher scores or percentages indicate worse symptoms. Points are estimated means from models with error bars representing 95% CIs. *P* value based on likelihood‐ratio test for overall comparison of treatments. [Colour figure can be viewed at wileyonlinelibrary.com]

**Fig. 3:**
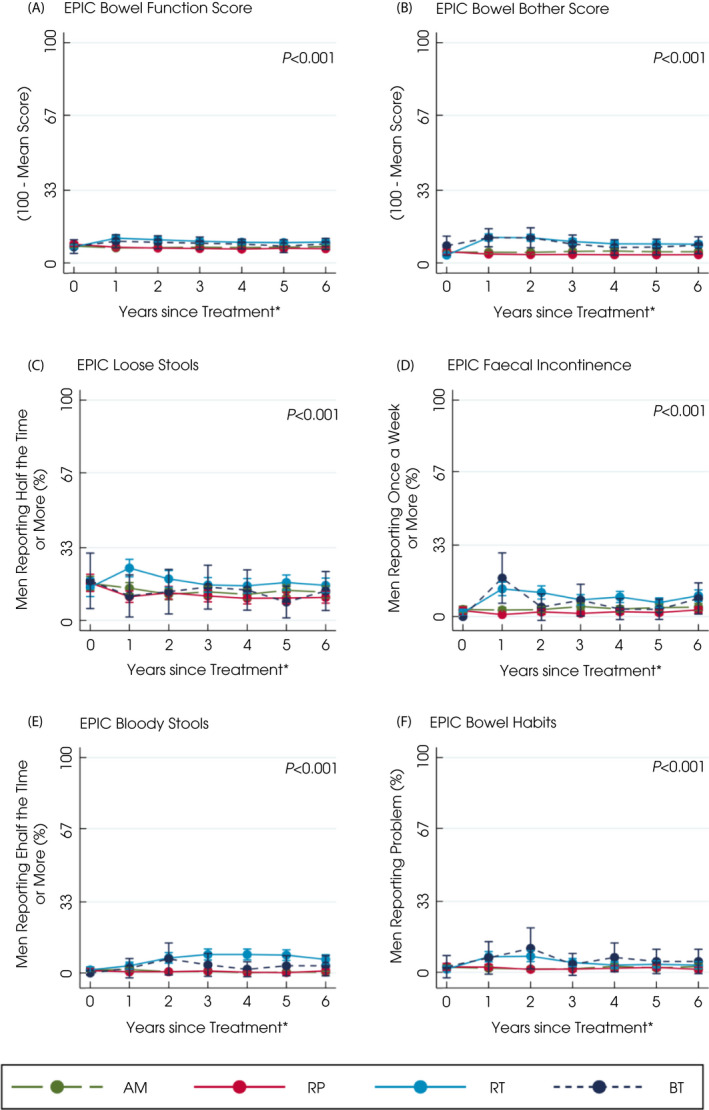
Patient‐reported bowel symptoms and QoL after primary treatments for localised prostate cancer over 6 years. *Defined as questionnaires completed for e.g., year 2 as between 1 and 2 years after treatment. Points are estimated means from models with error bars representing 95% CIs. Higher scores or percentages indicate worse symptoms. *P* value based on likelihood‐ratio test for overall comparison of treatments. [Colour figure can be viewed at wileyonlinelibrary.com]

Participant characteristics and symptoms were similar at baseline across all treatments. The median (interquartile range) age was 62 (50–73) years, with BT men on average 2 years younger [16] and 99% of men reported White ethnicity. Baseline symptoms were generally infrequent but included nocturia reported by 20% of men, (Fig. 1g, Table S2), increased daytime urinary frequency by 30% (Table S2), erectile dysfunction by 35%, (Fig. 2a, Table S3), loose stools by 17% (Fig. 3c, Table S4), and anxiety by 20% (Fig. S2 and Table S6).

### Active Monitoring

Men receiving and staying on AM experienced functional deteriorations likely due to ageing. Voiding symptoms and nocturia gradually increased to 38% of men by 6 years from 20% at baseline with no changes in the use of urinary pads, urination frequency, urinary summary, functional, bother scores or worsening impact of urinary QoL (Fig. 1a–g, Table S2). Erectile dysfunction increased gradually to affect 53% of men at 6 years and its QoL impact increased from 14% of men at baseline to 22% at 6 years. Overall sexual function, bother or sexual‐related QoL did not alter (Fig. 2a–e, Table S3) nor did bowel symptoms (overall summary, function, bother or QoL), or physical and mental health (Fig. S2a–f, Table S4).

### External‐beam RT and BT

Men receiving EBRT or BT experienced some changes in sexual, urinary and bowel function. Nocturia was reported by 42% of men immediately after EBRT and BT (43%) (*P* < 0.001 compared with RP or AM), which was increased from 20% at baseline but became comparable or less than AM by the sixth year (36% EBRT, 25% BT; Fig. 1g, Table S2). Men who received BT also reported more urinary voiding symptoms and higher irritative scores in the first year following treatment (EPIC irritative score and ICS*male*SF score; Fig. 1e, Table S2). Neither treatment affected urinary incontinence (1–3% of men reported pad use at 6 years; Fig. 1a–c, Table S2), urinary summary, functional, bother scores or QoL (Fig. 1d,f, Table S2). At 1 year after EBRT, 69% of men reported erectile dysfunction (compared with 38% of men at baseline), which was a moderate/big problem for 44% of men (20% of men at baseline). At 6 years, 74% of men still reported erectile dysfunction (problem for 39%) after EBRT compared with 53% for men on AM (problem for 26%, *P* < 0.001). In the first year following BT, 51% of men experienced erectile dysfunction (problem for 22%), which was comparable to AM by 6 years (52% of men and 29% a problem; Fig. 2a, Table S3). Sexual summary, function, bother and QoL scores were also better after BT than EBRT (Fig. 2c–e, Table S3). Bowel summary, function and bother scores and problems with symptoms worsened for 2 years after EBRT and BT (*P* < 0.001 with AM and RP; Fig. 3a,b,f, Table S4). Loose stools were most frequently experienced after EBRT (24% of men after 1 year and 19% by 2 years, *P* < 0.001 compared with other treatments; Fig. 3c, Table S4) and were still slightly higher than AM by 6 years (16% of men EBRT, 13% AM). Faecal incontinence increased after BT (reported by 18% of men) but declined to 8% by 6 years, whilst EBRT (13% of men at 1 year) remained higher than AM at 6 years (EBRT: 10% of men, AM: 4%; Fig. 3d, Table S4). Bloody stools were reported by 7% of men 2 years after EBRT, which continued over 6 years (6%; Fig. 3e, Table S4). Physical and mental health were unaffected by either treatment (Fig. S2a–d, Table S6).

### Radical Prostatectomy

Men receiving a RP experienced urinary incontinence and sexual dysfunction. In the first year after surgery 36% of men reported urinary leakage (at least 1 incontinence pad/day) and 17% reported that incontinence caused interference with daily life (Fig. 1b,c and Table S2) and still affected 20% of men at 6 years (problem for 13%, *P* < 0.001 compared with other treatments). The EPIC, ICIQ and ICS*male*SF incontinence scores and ICS*male*SF QoL scores showed similar differences between treatments (*P* < 0.001; Fig. 1a,f, Table S2). In the first year after a RP, increased daytime frequency of urination was reported by 41% of men (from 36% at baseline) and nocturia also increased to 27% of men (23% at baseline) but both had reduced at 6 years (daytime urination 33% of men, nocturia 22%, 38%, *P* < 0.001; Fig. 1g and Table S2). The EPIC urinary summary and function scores remained worse than other treatments throughout and bother scores were increased following surgery but were similar to AM by 6 years (Fig. 1d and Table S2). The EPIC urinary irritative score and ICS*male*SF voiding scores remained low over 6 years (Fig. 1e and Table S2). Erectile dysfunction affected 95% of men after surgery (35% at baseline) and was a moderate/big problem for 70% (17% at baseline) and continued for 85% of men at 6 years (problem for 50%, *P* < 0.001; Fig. 2a,b and Table S3). Similar decrements occurred in the sexual summary, function, bother and QoL scores (Fig. 2c–e and Table S3). Bowel function (Fig. 3a–f and Table S4), physical and mental health, anxiety or depression were unaltered following surgery (Fig. S2a–d, Table S6).

### Radical Treatments Following AM

Urinary leakage (at least daily pad use) was more frequent in men having surgery after AM (48%) compared with 36% initially, which persisted over 4 years (28% subsequently, 19% initially, *P* < 0.001; Fig. S3a, Table S5). Erectile dysfunction was frequently reported after immediate and later surgery with slightly more resolution over time (95% of men and 96% respectively immediately; 84% and 89% after 4 years, *P* = 0.033; Fig. S3c, Table S5). Nocturia and bloody stools showed no differences between immediate RP or surgery after AM (nocturia *P* = 0.277, bloody stools *P* = 0.478; Fig. S3b,d, Table S5). Depression was slightly worse following surgery after AM compared to immediate RP (*P* = 0.084; Fig. S4, Table S7) whereas anxiety, mental and physical health were comparable.

Nocturia was more frequently reported following EBRT after AM compared to immediate RT (52% of men subsequently, 42% immediately; at 4 years 41% and 33%, respectively, *P* = 0.087; Fig. S4b, Table S5,) as was erectile dysfunction (87% of men subsequently, 69% immediately; at 4 years 81% and 69%, respectively, *P* = 0.002; Fig. S4c, Table S5,). Urinary leakage (*P* = 0.911) and bloody stools (*P* = 0.907) were similar with immediate EBRT and after AM (Fig. S3a,b, Table S5). Mental health, anxiety and depression were comparable with immediate or delayed EBRT, whereas physical health was slightly worse after delayed EBRT than after immediate RT (Fig. S4, *P* = 0.02, Table S7).

### Treatment Impacts by Age

Some treatment impacts were greater in men aged 65–69 years at diagnosis compared with younger men (50–64 years, utilising interaction tests of age and symptoms in Table S8). Nocturia was worse in older men receiving AM (baseline: younger 15% compared with 29% older; 4 years 29% compared to 42%, Fig. S5b, Table S8) and following radical treatments (interaction *P* = 0.016; Fig. S5b, Table S8). Erectile dysfunction after EBRT or RP was similar for older and younger men over 6 years (interaction *P* = 0.198; Fig. S5c, Table S8). Daily incontinence pads were used by 42% of older men after surgery compared with 33% of younger men (interaction *P* = 0.474; Fig. S5a, Table S8). Bloody stools after EBRT were comparable across age groups (interaction *P* = 0.340; Fig. S5d, Table S8).

## Discussion

Men who make decisions about treatment for localised prostate cancer need to be able to assess the trade‐off between oncological benefits with treatment impacts on their QoL. Our report highlights radical treatment side‐effect profiles utilising PROMs over 6 years and deterioration in sexual and urinary function in men receiving AM. This analysis of PROMs related to the treatment actually *received* by ProtecT participants clearly shows the different impacts experienced over 6 years, without potential dilution by the ITT approach. Furthermore, larger numbers were analysed through combining men randomised with those who selected their treatment and were followed‐up over 6 years. Men remaining on AM experienced gradual declines in sexual and urinary function over time, and no change in bowel function or urinary incontinence. Impacts of radical treatments were more immediate and persistent. After RP, there was an immediate impact on sexual function with limited recovery and persistence of symptoms, worse than after other radical treatments. After RP, there was an immediate increase in urinary leakage requiring incontinence pads, with some improvement over the following few years, but persistent symptoms remaining in 20% of participants at 6 years. After EBRT with neoadjuvant ADT or BT, there was an immediate impact on sexual (particularly erectile) function that was less severe than for RP, and while this improved after 2 years with BT, the impact was sustained over time for EBRT, and was worse than in men receiving AM. Overall bowel function and bother were worse after EBRT and BT compared to AM or RP particularly for 2–3 years, with slightly lower scores for BT. Urinary voiding and nocturia were also adversely affected by BT and EBRT in the first year, but there was no impact on urinary continence. Mental and physical QoL were unaffected by any treatment. Overall, PROMs tended to be worse for older compared with younger men (<65 years).

The PROMs capture localised prostate treatment impacts effectively, but published results vary due to underlying differences in study designs, interventions, and measures [19, 20, 21]. In the randomised Scandinavian Prostate Cancer Group‐4 (SPCG‐4) trial [22], urinary pad utilisation was greater after surgery (41%) than on watchful waiting at 12 years but in both groups erectile dysfunction was >80%. Conversely, radical treatment impacts improved over 10 years in the Cancer of the Prostate Strategic Urologic Research Endeavor (CaPSURE) cohort (based on 15% of the cohort) [23]. The LAParoscopic Prostatectomy Robot Open study (LAPPRO) trial showed better erectile function at 24 months after robot‐assisted RP (RARP) compared with an open RP (based on the International Index of Erectile Function) and a small benefit for open RP regarding urinary leakage (based on the urinary PROM used in SPCG‐4) [24]. A systematic review of prostate cancer RT trials [25] concluded that BT had less impact on sexual function than EBRT with insufficient evidence on urinary function. A trial of hypofractionated and conventional RT showed that no differences in bowel bother, overall urinary or sexual bother over 5 years for men with intermediate‐risk prostate cancer [21]. A more recent trial of ultra‐ and hypofractionated RT for intermediate‐risk prostate cancer showed that the increased urinary and bowel bother did not persist over 4 years, but sexual bother increased over time in both groups [26].

The ProtecT results are based on a large and well‐characterised population‐based cohort with 6 years annual follow‐up, low attrition, and outcomes assessed at diagnosis, thus minimising recall bias. AM men were comparable to those undergoing radical treatments, unlike many other cohorts [20], and received standard clinical follow‐up without watchful waiting/observation patients who may have greater co‐morbidities. The censoring of subsequent radical treatments highlighted the likely age‐related declines for men on AM acting as a comparator for radical treatments and complementing the ITT analysis [4], where men receiving radical treatments were analysed in the AM group. Results from the 270 men undergoing radical treatments after a period of AM is also potentially novel. These validated PROMs included core outcomes [27] whereas the diversity of PROMs in RT trials for localised prostate cancer prevented the completion of meta‐analyses [6].

There are limitations to this study. The ProtecT participants were mostly of White ethnicity (broadly reflecting the recruiting centres populations) [8], so these results may not be informative for other ethnicities. Radical treatment techniques have evolved since the trial commenced, so questions were raised whether ProtecT reflected contemporary approaches such as RARP and newer methods of irradiation. To investigate the potential similarities between ProtecT and other cohorts, we compared contemporary treatments (IMRT low‐risk group patients and RARP) from the CEASAR [2] cohort, which used the EPIC‐26 (domain scores correlate with EPIC‐50 in ProtecT) [28] with EBRT and open RP in ProtecT (Table S9). Urinary leakage was slightly greater following IMRT (6%) than after EBRT (1%) with comparable irritative scores. Erectile dysfunction was similar between EBRT and IMRT, whilst bloody stools only occurred after EBRT (8%), likely due to higher rectal exposure. BT results were comparable in both studies. Urinary leakage was slightly higher after RARP (10%) than open RP (6%) and a greater problem (6% vs 12%). Conversely, erectile dysfunction was less frequent after RARP (61% vs 71% after open RP), likely due to improved neurovascular bundle preservation, although 24% of the RARP group were aged ≥70 years [29] (ProtecT maximum 69 years at recruitment). Active surveillance men reported greater urinary and sexual dysfunction in the CEASAR study, as 25% received radical treatments but censored in these ProtecT treatment‐received analyses. However, the CEASAR active surveillance protocol had different selection criteria and monitoring compared with ProtecT that might have affected PROMs.

In order to inform patient treatment decision making, these functional and QoL outcomes need to be considered alongside the small reduction in disease‐specific mortality from radical treatments compared with AM (ProtecT treatment‐received analysis) [17], and the Prostate cancer Intervention vs Observation Trial (PIVOT) [30] 12‐year follow‐up, which suggested an overall mortality benefit for surgery over watchful waiting in the intermediate‐risk disease group.

In summary, these full 6‐year functional and QoL profiles should inform treatment decision‐making for men with newly diagnosed localised prostate cancer and their treating clinicians. The long‐term side‐effects of radical treatments, as well as naturally deteriorating sexual and urinary function when on AM, need to be weighed against the potential oncological benefits of radical treatments.

## Disclosures of Interest

Ms Young: NIHR pre‐doctoral Fellowship. Professor Mason: Paid membership of Data Monitoring Committees for Clovis and Endocyte. Professor Martin: Receipt of Cancer Research UK grant at Univeristy of Bristol. The remaining authors have no disclosures.

## Author Contributions

Professors Donovan, Hamdy, Lane and Metcalfe and Neal had full access to the study data and take responsibility for the integrity of the data and the accuracy of the data analysis. Concept and design: Donovan, Hamdy, Neal. Acquisition, analysis, or interpretation of the data: Lane, Avery, Blazeby, Davis, Turner, Wade, Walsh. Drafting of the manuscript: Lane, Donovan, Hamdy, Metacalfe, Neal. Critical revision of the manuscript for important intellectual content: All authors. Statistical design and analysis: Metcalfe, Peters, Young. Obtained funding: Donovan, Hamdy, Neal.

## Funding

The study was supported by the NIHR Health Technology Assessment Programme (NIHR HTA: projects 96/20/06, 96/20/99, with University of Oxford as sponsor). Professor Lane is supported by the Bristol Randomised Trials Collaboration, a UKCRC registered Clinical Trials Unit (CTU) which, as part of the Bristol Trials Centre, is in receipt of NIHR CTU Support Funding. Professor Hamdy is supported by the Oxford NIHR Biomedical Research Centre Surgical Innovation and Evaluation Theme and Cancer Research UK Oxford Centre. Professors Blazeby, Lane and Martin and Dr Avery are supported by the NIHR Biomedical Research Centre at University Hospitals Bristol and Weston NHS Foundation Trust and the University of Bristol (BRC‐1215‐200011).

## Role of the Funder/Sponsor

The funder had no role in the study design, data collection, analysis, interpretation, writing of the paper or decision to submit for publication.

## Disclaimer

The views and opinions expressed in this article are those of the authors and do not necessarily reflect those of the UK Department of Health.

**Abbreviations**
ADT: androgen‐deprivation therapy
AM: active monitoring
BT: low‐dose‐rate brachytherapy
CEASAR: Comparative Effectiveness Analysis of Surgery and Radiation
(EB)(IM)RT: (external‐beam) (intensity‐modulated) radiotherapy
EPIC: Expanded Prostate Index Composite
HADS: Hospital Anxiety and Depression Scale
ICIQ: International Consultation on Incontinence Questionnaire
ITT: intention‐to‐treat
NIHR: UK National Institute of Health Research
PROMs: Patient‐reported outcome measures
ProtecT: Prostate Testing for Cancer and Treatment
QoL: quality of life
(RA)RP: (robot‐assisted) radical prostatectomy
SF‐12: Medical Outcomes Study 12‐Item Short Form Health Survey

## Supporting information


**Fig. S1** Primary and secondary treatments for localised prostate cancer received over 6 years of follow‐up.
**Fig. S2** Adjusted physical and mental health and psychological outcomes after primary treatments for prostate cancer over 6 years.
**Fig. S3** Patient‐reported urinary, bowel and sexual symptoms after radical treatment for localised prostate cancer, either initially, or after a period of AM over 4 years.
**Fig. S4** Adjusted QoL outcomes after receiving radical treatment after primary AM over 4 years.
**Fig. S5** Patient‐reported urinary, bowel and sexual symptoms by age groups by primary localised prostate cancer treatments over 6 years.
**Table S1** Response rates for exemplar PROMs in the treatment received analysis.
**Table S2** Adjusted urinary symptoms corresponding to Fig. 1 and subscales.
**Table S3** Adjusted sexual symptoms corresponding to Fig. 2 and subscales.
**Table S4** Adjusted bowel symptoms corresponding to Fig. 3 and subscales.
**Table S5** Adjusted symptoms from immediate radical treatments or after AM corresponding to Fig. S3.
**Table S6** Adjusted QoL items by treatment received corresponding to Fig. S2.
**Table S7** Adjusted QoL of radical treatments after AM and immediate: radical treatment corresponding to Fig. S4.
**Table S8** Adjusted interaction between treatment and age group on symptoms corresponding to Fig. S5.
**Table S9** Adjusted symptoms and quality 5 years after enrolment in the CEASAR cohort or diagnosis in the ProtecT trial.

## Data Availability

Individual participant data that underlie the results reported in this article, after de‐identification (text, tables, figures, and appendices) will be made available (the study protocol and statistical analysis plan is published). The data will be available to approved researchers following submission of a proposal (directed to f.c.hamdy@nds.ox.uk); to gain access, data requestors will need to sign a data access agreement.
