# Supplementary material for: Functional and quality of life outcomes of localised prostate cancer treatments (Prostate Testing for Cancer and Treatment [ProtecT] study)
Source: BJU Int. 2022 May 3;130(3):370–80. doi: 10.1111/bju.15739 (PMC9543725; doi:10.1111/bju.15739)
Supplement: Supplementary file 1 — Fig. S1 Primary and secondary treatments for localised prostate cancer received over 6 years of follow‐up. Fig. S2 Adjusted physical and mental health and psychological outcomes after primary treatments for prostate cancer over 6 years. Fig. S3 Patient‐reported urinary, bowel and sexual symptoms after radical treatment for localised prostate cancer, either initially, or after a period of AM over 4 years. Fig. S4 Adjusted QoL outcomes after receiving radical treatment after primary AM over 4 years. Fig. S5 Patient‐reported urinary, bowel and sexual symptoms by age groups by primary localised prostate cancer treatments over 6 years. Table S1 Response rates for exemplar PROMs in the treatment received analysis. Table S2 Adjusted urinary symptoms corresponding to Fig. 1 and subscales. Table S3 Adjusted sexual symptoms corresponding to Fig. 2 and subscales. Table S4 Adjusted bowel symptoms corresponding to Fig. 3 and subscales. Table S5 Adjusted symptoms from immediate radical treatments or after AM corresponding to Fig. S3. Table S6 Adjusted QoL items by treatment received corresponding to Fig. S2. Table S7 Adjusted QoL of radical treatments after AM and immediate: radical treatment corresponding to Fig. S4. Table S8 Adjusted interaction between treatment and age group on symptoms corresponding to Fig. S5. Table S9 Adjusted symptoms and quality 5 years after enrolment in the CEASAR cohort or diagnosis in the ProtecT trial. [file BJU-130-370-s001.pdf]

ProtecT Supplementary Online Content

Table of contents:

***Figure S1*. Primary and secondary treatments for localized prostate cancer received over 6 years of follow up 2**

***Figure S2*. Adjusted physical and mental health and psychological outcomes after primary treatments for prostate cancer over 6 years 3**

***Figure* S3. Patient-reported urinary, bowel and sexual symptoms after radical treatment for localised prostate cancer, either initially, or after a period of active monitoring over 4 years** 4

***Figure S4*. Adjusted quality of life outcomes after receiving radical treatment after primary active monitoring over 4 years............5**

***Figure S5.* Patient-reported urinary, bowel and sexual symptoms by age groups by primary localised prostate cancer treatments over 6 years............6**

***Table S1*. Response rates for exemplar PROMs in the treatment received analysis............7**

***Table S2*. Adjusted urinary symptoms corresponding to Figure 1 and subscales 8**

***Table S3*. Adjusted sexual symptoms corresponding to Figure 2 and subscales............** 10

***Table S4*. Adjusted bowel symptoms corresponding to Figure 3 and subscales............11**

***Table S5*. Adjusted symptoms from immediate radical treatments or after active monitoring corresponding to Figure S3 .... 13**

Table S6. Adjusted quality of life items by treatment received corresponding to Figure S2............ 14

***Table S7*. Adjusted quality of life of radical treatments after active monitoring and immediate: radical treatment corresponding to Figure S4 .... 15**

***Table S8*. Adjusted interaction between treatment and age group on symptoms corresponding to Supplementary Figure S5............ 16**

***Table S9*. Adjusted symptoms and quality five years after enrolment in the CEASAR cohort or diagnosis in the ProtecT trial 17**

***Figure S1*. Primary and secondary treatments for localized prostate cancer received over 6 years of follow up**

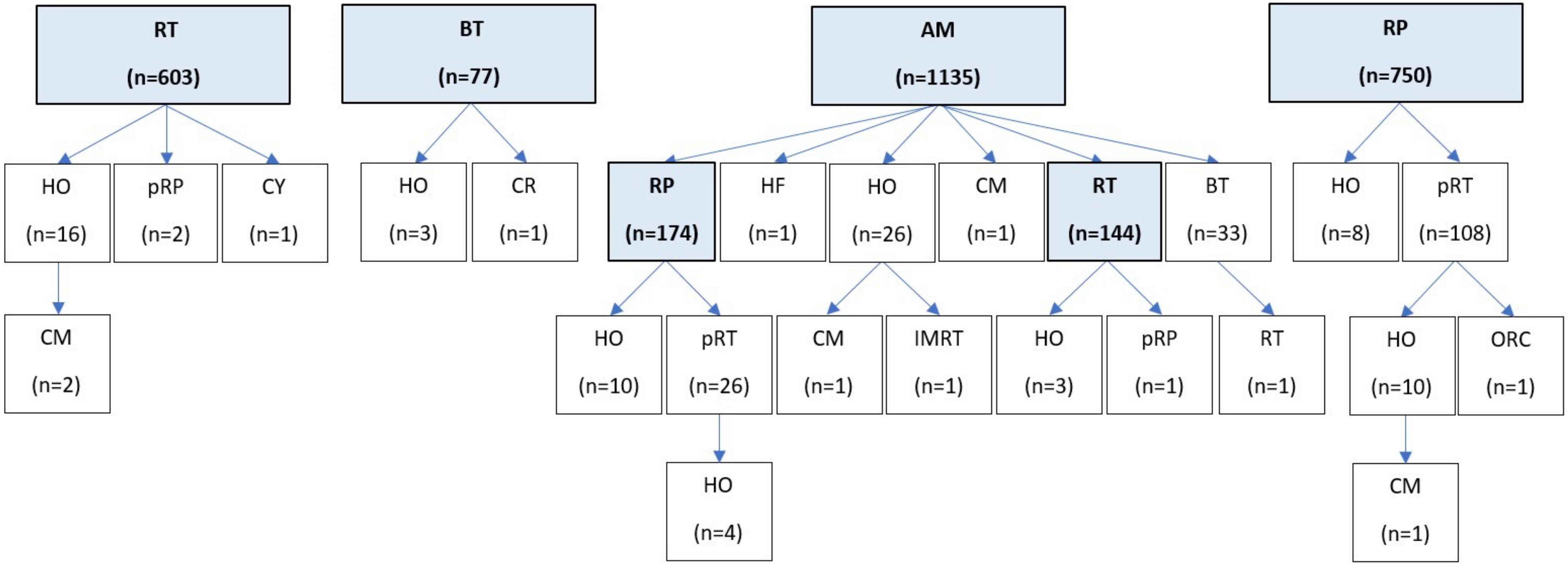

Abbreviations: RT=radiation, BT=brachytherapy, RP=radical prostatectomy, AM=active monitoring, HO=hormones, pRT=post-surgery radiotherapy, pRP=post-radiotherapy surgery, CM=chemotherapy, HF=HIFU, IMRT=intensity-modulated radiation therapy, ORC=subcapsular orchidectomy + CPA, CY=cystoprostatectomy (for bladder cancer), CR=cryotherapy
Groups highlighted in blue analysed with others until they received a new treatment.

***Figure S2*. Adjusted physical and mental health and psychological outcomes after primary treatments for prostate cancer over 6 years**

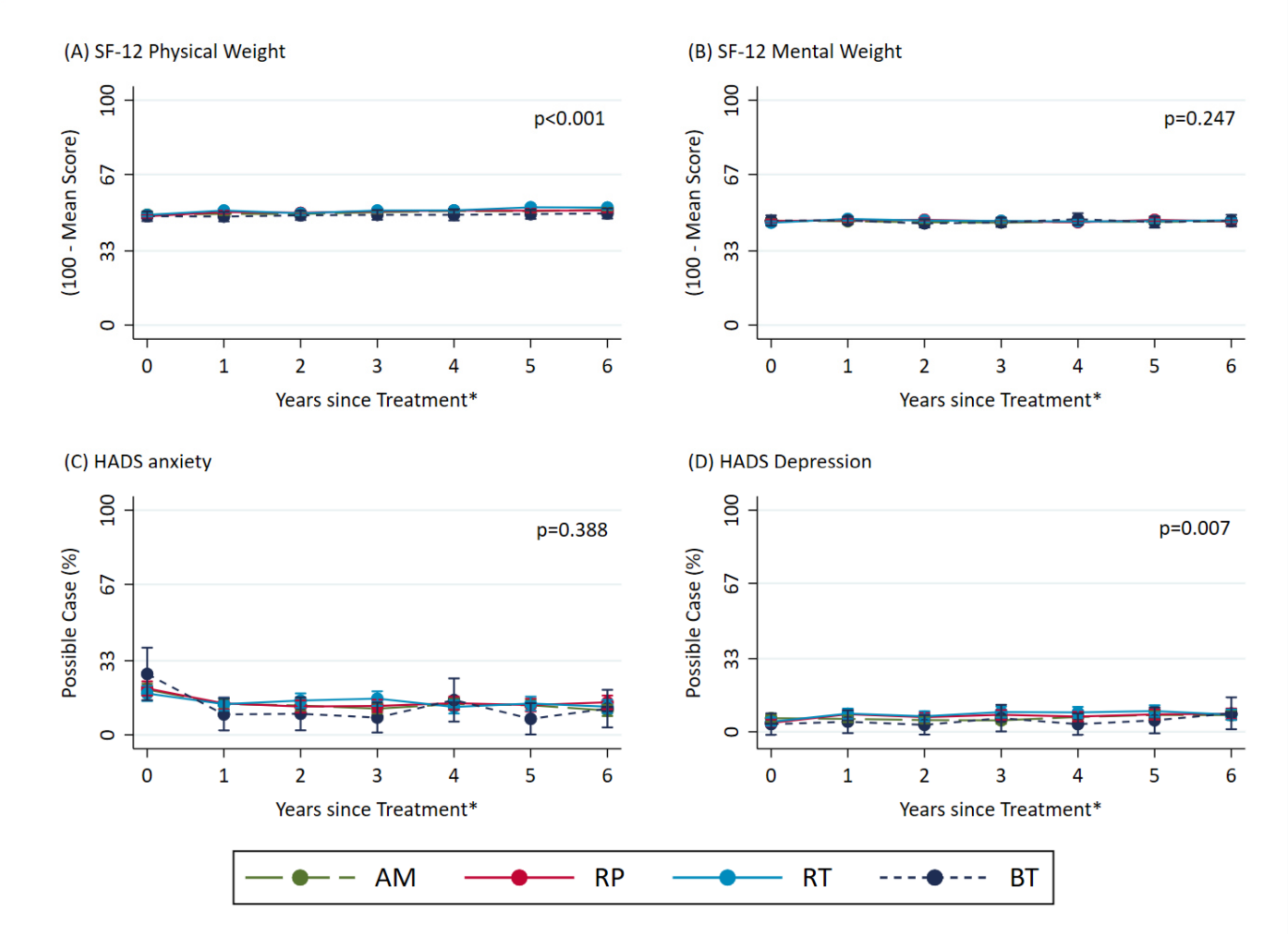

Abbreviations: AM, active monitoring, RP, radical prostatectomy, RT, radiation, BT brachytherapy. *Time from treatment is the time between diagnosis and start of radical treatment or radical treatment after active monitoring and defined as questionnaires completed for e.g. year 2 as between 1 and 2 years after treatment. Higher scores or percentages indicate worse symptoms with SF-12 symptoms reversed.

***Figure S3*. Patient-reported urinary, bowel and sexual symptoms after radical treatment for localised prostate cancer, either initially, or after a period of active monitoring over 4 years**

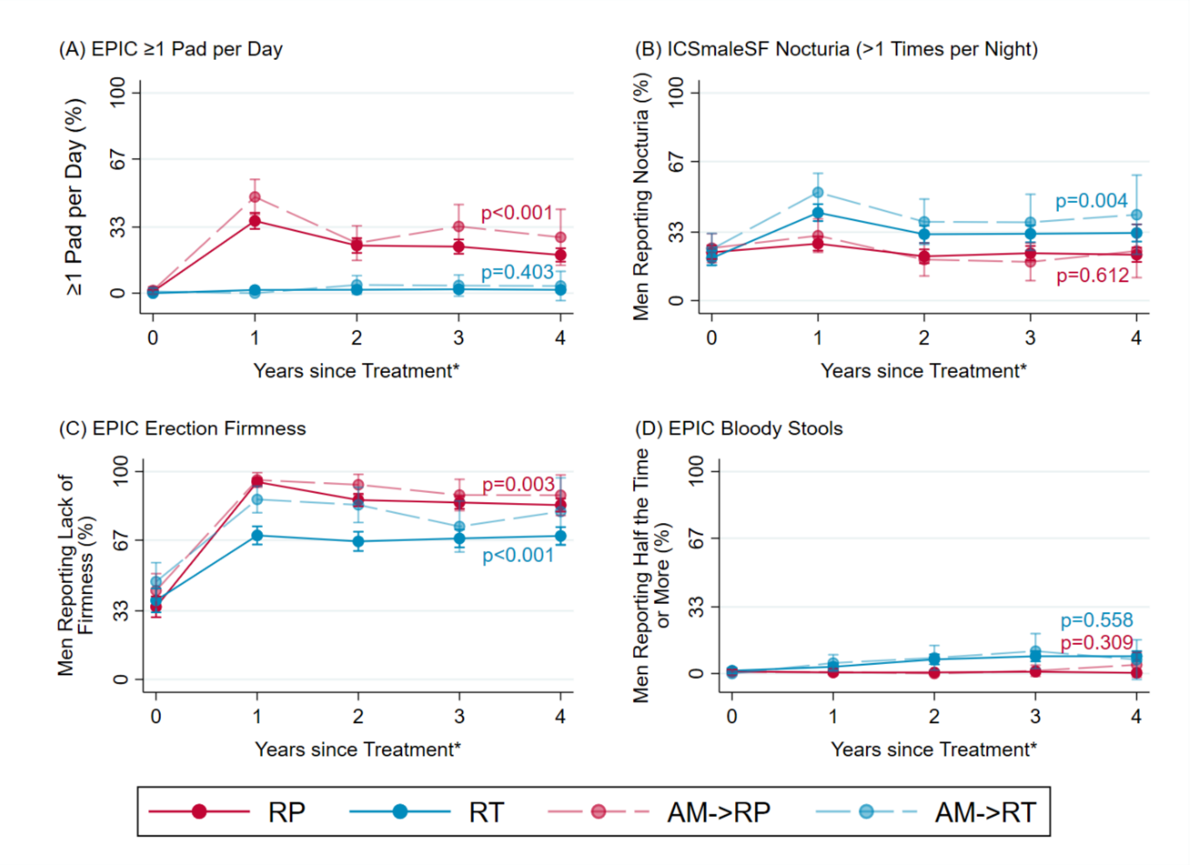

Abbreviations: AM, active monitoring; RP, radical prostatectomy; RT, EBRT. *Time from treatment is between diagnosis and radical treatment or radical treatment after active monitoring and for questionnaires completed for e.g. year 2 as between 1 and 2 years after treatment. Higher scores or percentages indicate worse symptoms. Points are estimated means from models with error bars representing 95% CIs. P-value based on likelihood ratio test in blue (EBRT) or red (RP)) comparing symptoms between those men starting treatment within a year of diagnosis with those delaying it until at least a year after diagnosis after receiving active monitoring.

***Figure S4*. Adjusted quality of life outcomes after receiving radical treatment after primary active monitoring over 4 years**

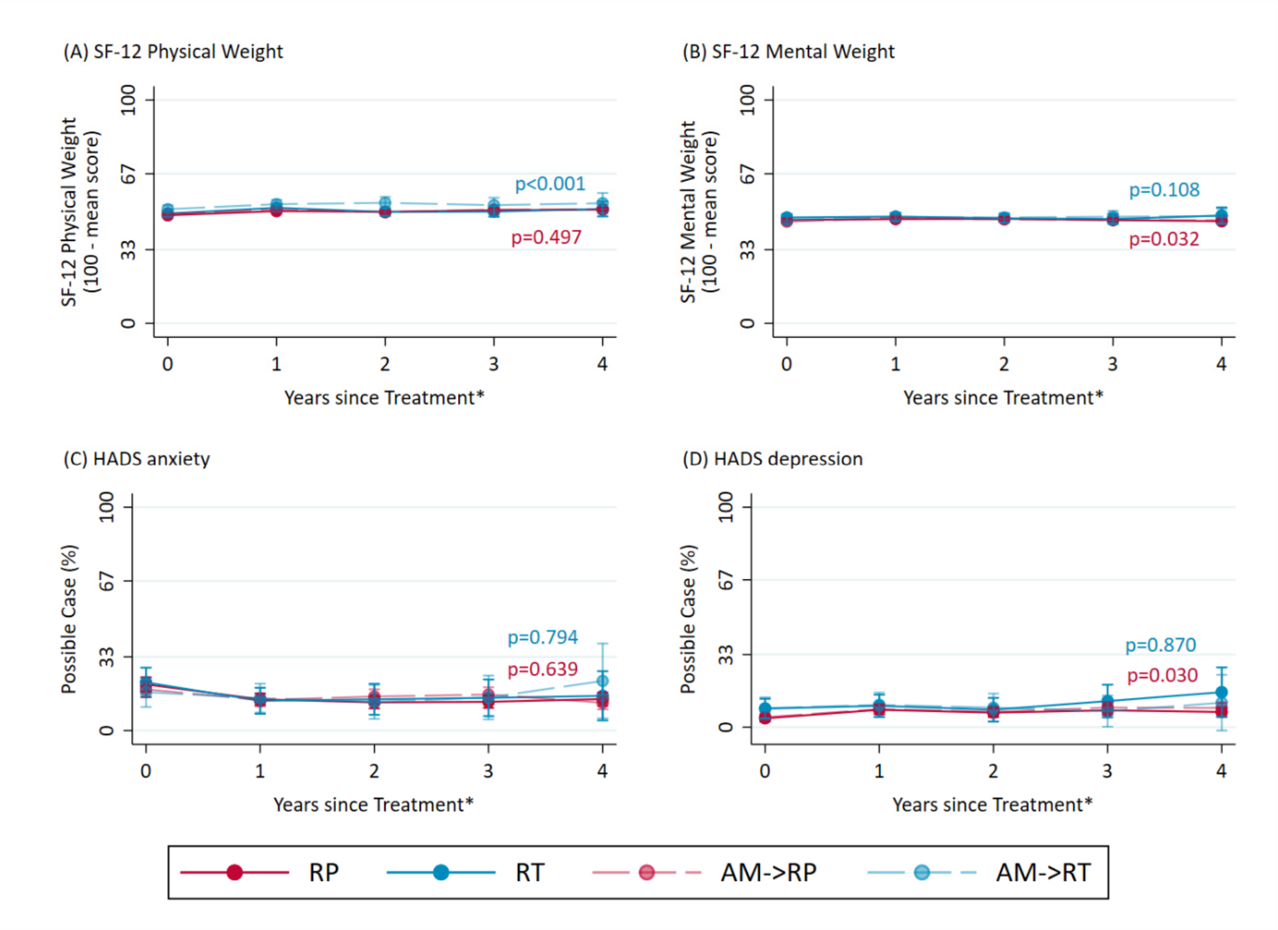

Abbreviations: AM, active monitoring, RP, radical prostatectomy, RT, radiation, BT brachytherapy. *Time from treatment is the time between diagnosis and start of radical treatment or radical treatment after active monitoring and defined as questionnaires completed for e.g. year 2 as between 1 and 2 years after treatment. Higher scores or percentages indicate worse symptoms with SF-12 symptoms reversed. P-value based on likelihood ratio test in blue (radiation) or red (radical prostatectomy) comparing symptoms between those men starting treatment within a year of diagnosis with those delaying it until at least a year after diagnosis after receiving active monitoring.

Figure S5. **Patient-reported urinary, bowel and sexual symptoms by age groups by primary localised prostate cancer treatments over 6 years**

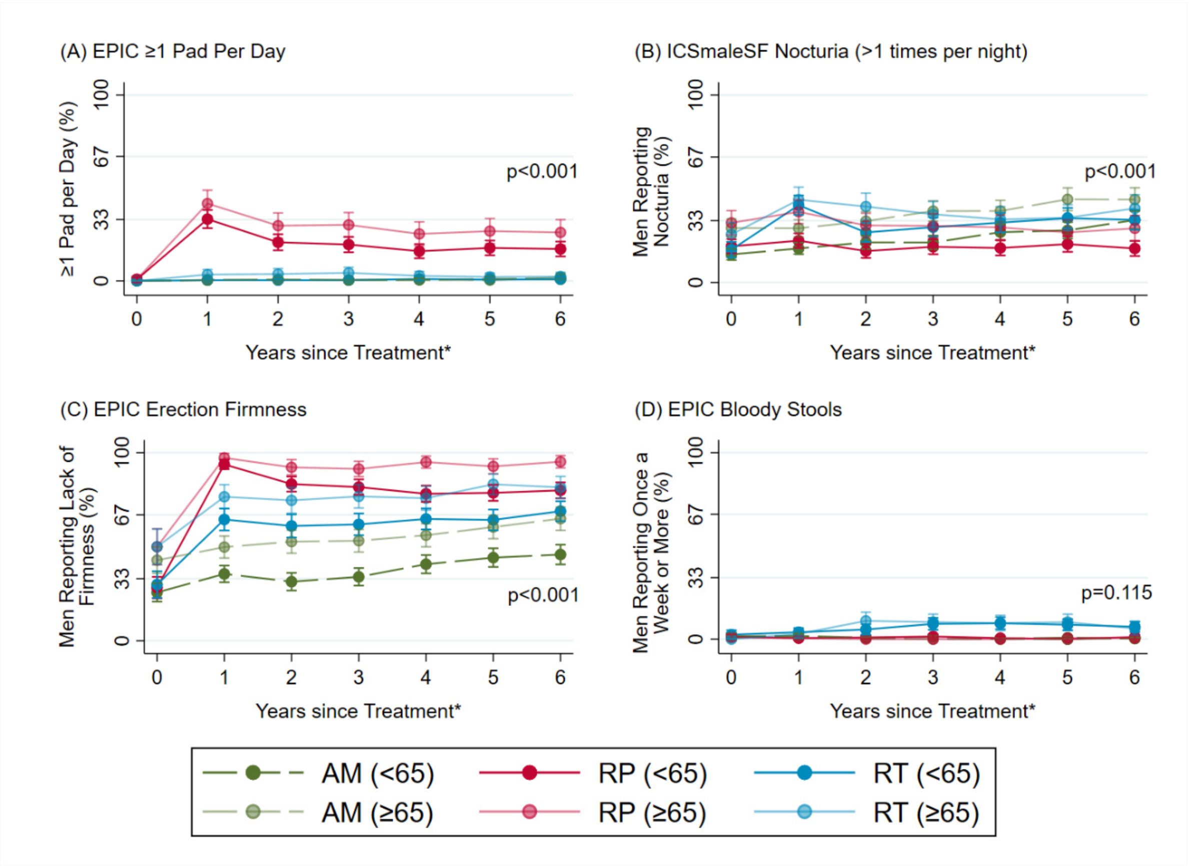

Abbreviations: AM, active monitoring; RP, radical prostatectomy; RT, EBRT; BT LDR brachytherapy. *Time from treatment between diagnosis and start of radical treatment. Includes those who have completed a questionnaire within the previous year, e.g. "2 Years from Treatment" refers to the period between 1 year after treatment and 2 years after treatment. Higher scores or percentages indicate more severe symptoms. Points are estimated means from models with error bars representing 95% CIs. P values are from a likelihood ratio test comparing a mixed effects regression model with an interaction (age*treatment) versus one without it.

***Table S1*. Response rates for exemplar PROMs in the treatment received analysis**

	**Completion rates: Completed[a]/expected[b] (%)**		
**Assessment period**	**ICIQ score[b]**	**EPIC sexual function score[c]**	**HADS anxiety**
Baseline	1807/2304 (78%)	970/1283 (76%)	2075/2560 (81%)
0-1 years	2158/2374 (91%)	1506/1658 (91%)	2274/2512 (91%)
1-2 years	1860/2372 (78%)	1424/1868 (76%)	1927/2410 (80%)
2-3 years	1820/2289 (80%)	1575/2002 (79%)	1871/2290 (82%)
3-4 years	1724/2175 (79%)	1595/2056 (78%)	1766/2175 (81%)
4-5 years	1671/2056 (81%)	1611/2059 (78%)	1708/2056 (83%)
5-6 years	1588/1955 (81%)	1566/2039 (77%)	1609/1955 (82%)

[a]Questionnaires completed within the time frame, excluding time points censored after second treatments and unexpected, [b]Expected counts were calculated if the questionnaire was introduced before the assessment period (excluding time points censored after second treatments, after death or after 15th November 2015), [b]ICIQ introduced 1st October 2001, [c]EPIC introduced 1st March 2005.

Table S2. Adjusted urinary symptoms corresponding to Figure 1 and subscales

Assessment period	AM		RP		RT		BT		P value[a]
	n (%) / *Mean (SD)*	N	n (%) / *Mean (SD)*	N	n (%) / *Mean (SD)*	N	n (%) / *Mean (SD)*	N	
ICIQ questionnaire									
**ICIQ Incontinence Score (0-21) – *corresponding to Figure 1A***									**<0.001**
Baseline	*1.10 (2.27)*	784	*1.28 (2.32)*	561	*1.16 (2.08)*	461	*1.31 (2.62)*	51	
0-1 years	*1.28 (2.36)*	960	*5.33 (4.59)*	674	*1.59 (2.52)*	545	*1.84 (2.86)*	62	
1-2 years	*1.38 (2.28)*	813	*4.46 (4.19)*	572	*1.71 (2.53)*	477	*1.73 (2.89)*	63	
2-3 years	*1.51 (2.44)*	768	*4.52 (4.13)*	555	*1.72 (2.62)*	495	*2.20 (3.33)*	61	
3-4 years	*1.65 (2.48)*	691	*4.32 (3.95)*	542	*1.91 (2.78)*	482	*1.90 (2.92)*	58	
4-5 years	*1.64 (2.56)*	657	*4.30 (3.90)*	525	*1.79 (2.69)*	475	*2.34 (3.63)*	58	
5-6 years	*1.72 (2.60)*	588	*4.41 (3.99)*	514	*1.82 (2.56)*	471	*2.17 (3.26)*	58	
**Incontinent (ICIQ score >0)**									**<0.001**
Baseline	200 (26%)	784	168 (30%)	561	139 (30%)	461	14 (27%)	51	
0-1 years	284 (30%)	960	556 (82%)	674	196 (36%)	545	22 (35%)	62	
1-2 years	277 (34%)	813	432 (76%)	572	186 (39%)	477	22 (35%)	63	
2-3 years	277 (36%)	768	426 (77%)	554	189 (38%)	495	26 (43%)	61	
3-4 years	266 (38%)	691	415 (77%)	542	208 (43%)	482	25 (43%)	58	
4-5 years	250 (38%)	657	405 (77%)	525	195 (41%)	475	25 (43%)	58	
5-6 years	226 (38%)	588	403 (78%)	514	205 (44%)	471	26 (45%)	58	
**ICIQ incontinence Problem (4+) – *corresponding to Figure 1C***									**<0.001**
Baseline	14 (2%)	790	12 (2%)	566	9 (2%)	466	3 (6%)	52	
0-1 years	21 (2%)	973	118 (17%)	680	13 (2%)	554	4 (6%)	64	
1-2 years	13 (2%)	824	76 (13%)	577	18 (4%)	483	3 (5%)	63	
2-3 years	14 (2%)	774	75 (13%)	562	16 (3%)	497	3 (5%)	62	
3-4 years	22 (3%)	701	60 (11%)	549	16 (3%)	483	2 (3%)	58	
4-5 years	21 (3%)	660	63 (12%)	530	21 (4%)	479	3 (5%)	60	
5-6 years	24 (4%)	592	66 (13%)	517	16 (3%)	474	4 (7%)	58	
ICS male questionnaire									
**Voiding score (0-20) – *corresponding to Figure 1E***									**<0.001**
Baseline	*3.24 (2.99)*	888	*3.43 (3.24)*	634	*3.16 (2.92)*	507	*3.17 (3.82)*	59	
0-1 years	*3.44 (3.14)*	998	*2.52 (2.89)*	686	*3.67 (3.41)*	562	*5.55 (3.89)*	67	
1-2 years	*3.68 (3.22)*	847	*2.29 (2.79)*	591	*3.14 (3.01)*	488	*4.31 (3.50)*	64	
2-3 years	*3.95 (3.36)*	797	*2.58 (2.89)*	576	*3.29 (3.05)*	508	*3.79 (3.58)*	66	
3-4 years	*4.08 (3.41)*	720	*2.43 (2.62)*	557	*3.27 (2.97)*	492	*3.55 (2.97)*	60	
4-5 years	*4.32 (3.46)*	678	*2.54 (2.74)*	543	*3.52 (3.17)*	488	*3.68 (3.19)*	60	
5-6 years	*4.43 (3.54)*	606	*2.64 (2.63)*	524	*3.43 (2.92)*	482	*3.73 (3.17)*	59	
**Incontinence score (0-24)**									**<0.001**
Baseline	*1.71 (1.80)*	887	*1.85 (1.87)*	634	*1.76 (1.82)*	506	*1.83 (2.22)*	59	
0-1 years	*1.68 (1.84)*	995	*4.60 (3.68)*	681	*2.12 (2.05)*	561	*2.16 (1.78)*	67	
1-2 years	*1.80 (1.94)*	844	*4.19 (3.52)*	582	*2.07 (2.01)*	489	*2.00 (1.93)*	64	
2-3 years	*1.96 (2.05)*	791	*4.35 (3.49)*	568	*2.17 (2.13)*	503	*2.24 (2.37)*	63	
3-4 years	*2.02 (2.09)*	710	*4.07 (3.27)*	552	*2.21 (2.21)*	490	*1.82 (1.72)*	60	
4-5 years	*2.11 (2.07)*	676	*4.23 (3.28)*	536	*2.18 (2.15)*	483	*2.32 (2.16)*	59	
5-6 years	*2.14 (2.16)*	605	*4.08 (3.15)*	525	*2.24 (2.18)*	478	*2.30 (2.16)*	60	
**ICSmale QoL ("somewhat/a lot" of impact) – *corresponding to Figure 1F***									**<0.001**
Baseline	29 (3%)	897	19 (3%)	636	13 (3%)	511	3 (5%)	59	
0-1 years	20 (2%)	1003	91 (13%)	689	31 (6%)	563	9 (13%)	67	
1-2 years	25 (3%)	842	58 (10%)	590	15 (3%)	488	5 (8%)	64	
2-3 years	32 (4%)	791	63 (11%)	574	18 (4%)	504	5 (8%)	64	
3-4 years	31 (4%)	714	45 (8%)	559	20 (4%)	495	3 (5%)	60	
4-5 years	35 (5%)	674	44 (8%)	541	24 (5%)	487	5 (8%)	60	
5-6 years	32 (5%)	606	44 (8%)	528	22 (5%)	478	5 (8%)	60	
**Daytime frequency (more frequent than once every three hours)**									**=0.293**
Baseline	265 (30%)	888	229 (36%)	631	162 (32%)	502	12 (20%)	59	
0-1 years	312 (31%)	1000	280 (41%)	685	202 (36%)	556	29 (44%)	66	
1-2 years	255 (30%)	840	194 (33%)	586	154 (31%)	489	18 (29%)	63	
2-3 years	247 (31%)	790	193 (34%)	572	165 (33%)	503	21 (33%)	64	
3-4 years	224 (31%)	713	185 (34%)	550	162 (33%)	494	22 (37%)	60	
4-5 years	212 (32%)	670	190 (36%)	534	152 (31%)	483	19 (32%)	60	
5-6 years	194 (32%)	601	173 (33%)	519	159 (33%)	479	14 (24%)	59	
**Nocturia (>1 times per night) – *corresponding to Figure 1G***									**<0.001**
Baseline	183 (20%)	894	147 (23%)	631	104 (21%)	507	9 (15%)	59	

0-1 years	223 (22%)	998	188 (27%)	685	239 (42%)	564	29 (43%)	67	
1-2 years	219 (26%)	844	126 (21%)	590	156 (32%)	488	19 (30%)	64	
2-3 years	221 (28%)	790	131 (23%)	573	161 (32%)	500	20 (32%)	63	
3-4 years	224 (31%)	715	123 (22%)	556	162 (33%)	497	16 (28%)	58	
4-5 years	230 (34%)	672	121 (22%)	538	166 (34%)	482	18 (30%)	60	
5-6 years	227 (38%)	601	114 (22%)	525	171 (36%)	479	15 (25%)	59	
EPIC questionnaire									
**≥1 Pad per day – *corresponding to Figure 1B***									**<0.001**
Baseline	0 (0%)	520	3 (1%)	362	0 (0%)	277	0 (0%)	39	
0-1 years	3 (0%)	756	197 (36%)	546	7 (2%)	442	1 (2%)	46	
1-2 years	4 (1%)	660	123 (24%)	517	7 (2%)	414	2 (4%)	45	
2-3 years	3 (0%)	691	126 (23%)	542	9 (2%)	466	2 (4%)	54	
3-4 years	4 (1%)	655	102 (19%)	535	8 (2%)	478	3 (5%)	56	
4-5 years	5 (1%)	646	109 (21%)	529	6 (1%)	485	3 (5%)	57	
5-6 years	8 (1%)	595	101 (20%)	505	7 (1%)	475	2 (3%)	59	
**Urinary summary score (0-100, reversed[b]) – *corresponding to Figure 1D***									**<0.001**
Baseline	*6.62 (8.52)*	520	*7.38 (9.21)*	362	*7.06 (8.69)*	276	*8.19 (10.23)*	39	
0-1 years	*6.99 (8.40)*	756	*15.70 (14.58)*	546	*9.49 (10.85)*	443	*14.72 (12.52)*	46	
1-2 years	*6.97 (8.27)*	659	*12.25 (12.64)*	516	*7.90 (8.85)*	413	*13.57 (13.84)*	45	
2-3 years	*7.78 (9.00)*	693	*12.47 (12.34)*	541	*8.47 (10.06)*	459	*12.56 (14.16)*	54	
3-4 years	*8.28 (9.56)*	654	*11.27 (11.43)*	536	*8.20 (9.48)*	477	*9.48 (11.41)*	56	
4-5 years	*8.25 (9.31)*	644	*11.48 (11.50)*	529	*8.23 (9.47)*	483	*10.72 (13.29)*	56	
5-6 years	*8.96 (9.80)*	594	*11.11 (11.10)*	508	*8.23 (8.95)*	475	*9.45 (11.19)*	59	
**Urinary function score (0-100, reversed[b])**									**<0.001**
Baseline	*4.16 (7.41)*	520	*4.58 (8.61)*	363	*4.74 (7.60)*	278	*3.75 (7.01)*	39	
0-1 years	*4.92 (7.86)*	758	*18.55 (16.09)*	549	*6.29 (8.70)*	442	*9.90 (10.19)*	46	
1-2 years	*4.56 (7.16)*	660	*14.28 (13.83)*	518	*6.09 (8.23)*	414	*9.91 (11.33)*	45	
2-3 years	*5.23 (7.68)*	693	*14.68 (13.85)*	542	*5.89 (8.51)*	465	*9.58 (11.61)*	54	
3-4 years	*5.59 (8.05)*	656	*13.29 (12.82)*	538	*6.19 (8.70)*	478	*8.01 (10.90)*	56	
4-5 years	*5.69 (8.41)*	647	*13.65 (13.08)*	531	*5.88 (8.08)*	484	*7.81 (11.21)*	57	
5-6 years	*6.09 (8.60)*	595	*13.35 (13.10)*	510	*5.95 (7.67)*	475	*7.94 (9.74)*	59	
**Urinary bother score (0-100, reversed[b])**									**=0.001**
Baseline	*8.38 (11.11)*	520	*9.38 (11.72)*	364	*8.67 (11.19)*	275	*11.36 (13.61)*	39	
0-1 years	*8.50 (10.53)*	753	*13.62 (15.30)*	546	*11.84 (14.02)*	443	*18.17 (16.73)*	46	
1-2 years	*8.70 (10.74)*	657	*10.78 (13.86)*	513	*9.20 (11.06)*	410	*16.19 (17.17)*	45	
2-3 years	*9.64 (11.70)*	694	*10.85 (13.23)*	542	*10.15 (12.59)*	458	*14.68 (18.07)*	54	
3-4 years	*10.17 (12.31)*	653	*9.79 (12.17)*	532	*9.63 (11.87)*	476	*10.52 (13.91)*	56	
4-5 years	*10.06 (11.71)*	645	*9.92 (12.22)*	527	*9.87 (12.19)*	481	*12.69 (16.26)*	56	
5-6 years	*11.03 (12.60)*	592	*9.46 (11.45)*	507	*9.90 (11.51)*	476	*10.53 (13.70)*	59	
**Urinary incontinence score (0-100, reversed[b])**									**<0.001**
Baseline	*5.95 (10.76)*	513	*6.38 (10.86)*	358	*6.86 (11.02)*	273	*5.49 (10.50)*	39	
0-1 years	*7.18 (11.33)*	750	*28.50 (24.93)*	533	*8.55 (11.94)*	438	*10.61 (13.75)*	46	
1-2 years	*7.04 (11.02)*	649	*21.89 (21.62)*	502	*9.51 (12.59)*	408	*9.61 (13.79)*	45	
2-3 years	*7.86 (11.43)*	681	*22.96 (22.14)*	529	*9.02 (12.32)*	451	*11.22 (16.99)*	53	
3-4 years	*8.25 (11.94)*	638	*20.69 (20.19)*	527	*9.44 (12.72)*	474	*11.58 (15.86)*	56	
4-5 years	*8.42 (12.31)*	636	*21.29 (20.95)*	521	*8.77 (11.88)*	472	*12.50 (18.10)*	56	
5-6 years	*9.03 (12.29)*	587	*20.80 (20.45)*	500	*9.25 (12.06)*	464	*10.56 (14.82)*	59	
**Urinary irritative score (0-100, reversed[b])**									**<0.001**
Baseline	*6.66 (8.60)*	520	*7.42 (9.45)*	362	*6.79 (8.52)*	276	*8.61 (10.35)*	39	
0-1 years	*6.45 (8.07)*	756	*7.78 (10.13)*	546	*9.11 (11.49)*	442	*16.07 (14.65)*	46	
1-2 years	*6.50 (8.22)*	659	*6.14 (8.93)*	517	*6.49 (8.01)*	413	*14.68 (15.18)*	45	
2-3 years	*7.19 (8.76)*	693	*5.99 (8.15)*	541	*7.56 (9.57)*	460	*12.37 (15.10)*	54	
3-4 years	*7.74 (9.37)*	654	*5.20 (7.39)*	536	*6.93 (8.61)*	477	*7.46 (9.57)*	56	
4-5 years	*7.59 (8.75)*	645	*5.50 (7.23)*	530	*7.29 (9.13)*	484	*8.93 (11.00)*	56	
5-6 years	*8.24 (9.54)*	594	*5.20 (6.94)*	508	*7.07 (8.32)*	475	*8.17 (10.34)*	59	

Abbreviations: AM=Active Monitoring, RP=Radical Prostatectomy, RT=Radiotherapy, BT=Brachytherapy, [a]P value from a likelihood ratio test comparing a mixed effects model, with and without treatment received as a covariate. Other covariates included a treatment cohort indicator (randomised/preference) and propensity scores. Baseline measures were not included, or adjusted for, based on this being an 'as treated' analysis. [b]Scores were reversed so that higher scores indicate worse symptoms

Table S3. Adjusted sexual symptoms corresponding to Figure 2 and subscales

Assessment period	AM		RP		RT		BT		P value[a]
	n (%) / *Mean (SD)*	N	n (%) / *Mean (SD)*	N	n (%) / *Mean (SD)*	N	n (%) / *Mean (SD)*	N	
EPIC questionnaire									
**Men reporting lack of firmness – *corresponding to Figure 2A***									**<0.001**
Baseline	164 (32%)	507	123 (35%)	352	105 (38%)	276	14 (37%)	38	
0-1 years	302 (41%)	735	514 (95%)	541	300 (69%)	433	23 (51%)	45	
1-2 years	255 (40%)	637	435 (86%)	504	267 (66%)	402	27 (61%)	44	
2-3 years	279 (42%)	670	452 (85%)	531	308 (68%)	454	29 (54%)	54	
3-4 years	302 (47%)	646	448 (84%)	534	319 (69%)	462	34 (60%)	57	
4-5 years	319 (50%)	632	438 (83%)	527	336 (71%)	470	34 (58%)	59	
5-6 years	310 (53%)	580	435 (85%)	512	346 (74%)	470	30 (52%)	58	
**Men reporting problem (moderate/big) with erectile dysfunction – *corresponding to Figure 2B***									**<0.001**
Baseline	70 (14%)	501	61 (17%)	353	55 (20%)	277	4 (11%)	38	
0-1 years	143 (20%)	725	378 (70%)	540	186 (44%)	422	10 (22%)	46	
1-2 years	123 (19%)	638	310 (62%)	504	149 (38%)	396	19 (42%)	45	
2-3 years	143 (22%)	659	274 (52%)	528	184 (41%)	448	17 (33%)	52	
3-4 years	147 (23%)	639	284 (53%)	532	176 (38%)	460	18 (31%)	58	
4-5 years	152 (24%)	628	259 (50%)	523	186 (40%)	470	13 (22%)	59	
5-6 years	146 (26%)	572	252 (50%)	509	181 (39%)	459	17 (29%)	59	
**Sexual summary score (0-100, reversed[b])**									**<0.001**
Baseline	*36.79 (20.91)*	496	*38.75 (23.32)*	347	*40.64 (25.32)*	273	*36.68 (19.80)*	37	
0-1 years	*40.86 (23.70)*	717	*76.12 (18.92)*	533	*62.17 (27.62)*	424	*49.00 (23.46)*	45	
1-2 years	*41.27 (23.86)*	630	*69.42 (21.71)*	499	*57.91 (25.49)*	390	*55.09 (24.27)*	45	
2-3 years	*42.67 (24.19)*	650	*68.87 (22.24)*	519	*59.56 (25.46)*	444	*53.30 (26.01)*	52	
3-4 years	*45.12 (24.66)*	632	*67.65 (23.06)*	527	*58.96 (25.61)*	454	*52.40 (25.56)*	56	
4-5 years	*45.90 (24.48)*	618	*67.66 (23.47)*	518	*60.62 (24.59)*	465	*50.92 (23.91)*	59	
5-6 years	*47.06 (24.17)*	565	*68.23 (22.88)*	507	*60.51 (24.93)*	452	*50.24 (23.85)*	58	
**Sexual function score (0-100, reversed[b]) – *corresponding to Figure 2C***									**<0.001**
Baseline	*44.07 (21.11)*	498	*45.70 (22.85)*	348	*48.26 (25.08)*	273	*43.17 (20.55)*	37	
0-1 years	*48.29 (23.95)*	722	*82.44 (17.74)*	534	*69.25 (27.21)*	425	*56.49 (24.98)*	45	
1-2 years	*48.61 (23.91)*	628	*75.77 (21.49)*	501	*64.38 (25.19)*	392	*60.42 (23.34)*	45	
2-3 years	*49.75 (24.23)*	657	*76.17 (21.84)*	526	*66.88 (25.17)*	450	*60.37 (25.61)*	54	
3-4 years	*52.46 (24.41)*	641	*75.41 (23.06)*	531	*66.44 (25.47)*	459	*60.02 (26.90)*	57	
4-5 years	*53.00 (24.42)*	626	*76.07 (23.26)*	518	*67.72 (24.46)*	471	*58.92 (24.71)*	59	
5-6 years	*55.33 (24.79)*	578	*77.01 (22.79)*	505	*68.69 (25.34)*	461	*58.01 (26.00)*	58	
**Sexual bother score (0-100, reversed[b]) – *corresponding to Figure 2D***									**<0.001**
Baseline	*20.10 (26.47)*	496	*23.07 (29.66)*	347	*24.14 (31.86)*	270	*21.55 (23.91)*	38	
0-1 years	*24.74 (29.69)*	716	*61.94 (32.29)*	534	*46.50 (37.78)*	422	*32.74 (29.67)*	46	
1-2 years	*25.18 (29.66)*	634	*55.29 (32.99)*	500	*43.57 (34.78)*	390	*41.57 (31.25)*	43	
2-3 years	*27.39 (30.69)*	655	*52.31 (34.90)*	520	*43.91 (35.33)*	441	*39.42 (33.66)*	52	
3-4 years	*28.91 (31.85)*	634	*50.43 (34.71)*	527	*42.52 (35.06)*	456	*34.54 (31.25)*	57	
4-5 years	*29.70 (31.14)*	623	*49.11 (35.83)*	518	*44.17 (35.38)*	464	*33.16 (30.14)*	59	
5-6 years	*29.55 (31.57)*	569	*49.04 (35.14)*	503	*42.51 (35.66)*	454	*32.20 (29.95)*	59	
**Men reporting impact (moderate/big) of sexual dysfunction on quality of life – *corresponding to Figure 2E***									**<0.001**
Baseline	69 (14%)	500	57 (16%)	351	46 (17%)	274	4 (11%)	38	
0-1 years	134 (19%)	721	341 (64%)	537	179 (42%)	426	10 (22%)	46	
1-2 years	117 (18%)	639	271 (54%)	502	155 (39%)	398	17 (39%)	44	
2-3 years	125 (19%)	657	249 (48%)	523	169 (38%)	447	16 (31%)	52	
3-4 years	133 (21%)	638	238 (45%)	531	165 (36%)	459	14 (25%)	57	
4-5 years	141 (22%)	627	242 (46%)	524	168 (36%)	469	12 (20%)	59	
5-6 years	124 (22%)	572	217 (43%)	509	168 (37%)	460	15 (25%)	59	

Abbreviations: AM=Active Monitoring, RP=Radical Prostatectomy, RT=Radiotherapy, BT=Brachytherapy, [a]P value from a likelihood ratio test comparing a mixed effects model, with and without treatment received as a covariate. Other covariates included a treatment cohort indicator (randomised/preference) and propensity scores. Baseline measures were not included, or adjusted for, based on this being an 'as treated' analysis.
[b]Scores were reversed so that higher scores indicate worse symptoms

Table S4. Adjusted bowel symptoms corresponding to Figure 3 and subscales

Assessment period	AM		RP		RT		BT		P value[a]
	n (%) / *Mean (SD)*	N	n (%) / *Mean (SD)*	N	n (%) / *Mean (SD)*	N	n (%) / *Mean (SD)*	N	
EPIC questionnaire									
**Bowel summary score**									**<0.001**
Baseline	*6.22 (7.75)*	517	*6.82 (8.34)*	362	*5.39 (6.93)*	276	*7.60 (10.59)*	39	
0-1 years	*5.87 (8.37)*	755	*5.59 (7.39)*	546	*11.42 (13.91)*	445	*10.63 (10.85)*	45	
1-2 years	*5.86 (8.27)*	657	*5.30 (7.36)*	516	*10.99 (12.07)*	415	*10.41 (11.31)*	45	
2-3 years	*6.20 (9.47)*	683	*5.18 (6.92)*	544	*9.78 (11.21)*	464	*8.63 (11.51)*	53	
3-4 years	*6.23 (9.49)*	657	*5.00 (7.10)*	536	*9.02 (11.19)*	475	*7.79 (10.01)*	58	
4-5 years	*6.13 (8.97)*	647	*5.15 (7.43)*	535	*8.91 (9.93)*	481	*7.41 (12.04)*	60	
5-6 years	*6.23 (9.40)*	591	*5.08 (6.78)*	520	*9.10 (10.70)*	475	*8.33 (11.06)*	60	
**Bowel function score – *corresponding to Figure 3A***									**<0.001**
Baseline	*7.66 (8.42)*	517	*8.50 (8.71)*	364	*7.25 (7.79)*	279	*7.42 (9.60)*	39	
0-1 years	*6.85 (8.50)*	758	*7.13 (8.17)*	548	*11.23 (12.43)*	446	*9.84 (9.79)*	45	
1-2 years	*6.94 (8.24)*	657	*6.77 (7.68)*	518	*10.54 (10.00)*	415	*9.38 (9.82)*	45	
2-3 years	*7.18 (9.38)*	685	*6.54 (7.27)*	543	*9.83 (10.01)*	465	*8.96 (10.07)*	53	
3-4 years	*7.00 (8.80)*	660	*6.28 (7.54)*	538	*9.38 (9.99)*	477	*8.56 (8.51)*	58	
4-5 years	*7.14 (9.23)*	651	*6.62 (7.66)*	537	*9.20 (9.14)*	481	*7.62 (11.38)*	60	
5-6 years	*7.37 (9.07)*	591	*6.44 (7.21)*	522	*9.50 (9.87)*	477	*8.63 (9.67)*	60	
**Bowel bother score – *corresponding to Figure 3B***									**<0.001**
Baseline	*4.87 (9.50)*	519	*5.09 (10.15)*	363	*3.56 (7.73)*	276	*7.78 (13.56)*	39	
0-1 years	*4.87 (9.84)*	756	*4.03 (8.35)*	547	*11.58 (17.09)*	445	*11.43 (13.65)*	45	
1-2 years	*4.78 (9.93)*	659	*3.81 (8.69)*	518	*11.42 (16.27)*	416	*11.43 (15.03)*	45	
2-3 years	*5.25 (11.06)*	691	*3.83 (8.44)*	546	*9.73 (14.30)*	466	*8.57 (15.08)*	55	
3-4 years	*5.42 (11.57)*	662	*3.72 (8.26)*	538	*8.68 (14.10)*	479	*7.02 (13.43)*	58	
4-5 years	*5.07 (10.17)*	653	*3.69 (8.81)*	537	*8.65 (13.10)*	488	*7.20 (13.82)*	60	
5-6 years	*5.09 (11.18)*	599	*3.74 (8.27)*	521	*8.55 (13.47)*	482	*8.04 (14.77)*	60	
**Loose stools – *corresponding to Figure 3C***									**<0.001**
Baseline	87 (17%)	517	62 (17%)	364	42 (15%)	280	7 (18%)	39	
0-1 years	111 (15%)	759	59 (11%)	549	106 (24%)	446	5 (11%)	45	
1-2 years	78 (12%)	657	65 (13%)	518	78 (19%)	414	6 (13%)	46	
2-3 years	89 (13%)	688	60 (11%)	543	75 (16%)	466	8 (15%)	53	
3-4 years	78 (12%)	659	54 (10%)	539	75 (16%)	478	8 (14%)	58	
4-5 years	89 (14%)	653	55 (10%)	541	83 (17%)	482	5 (8%)	60	
5-6 years	77 (13%)	598	55 (10%)	526	76 (16%)	478	8 (13%)	60	
**Faecal incontinence – *corresponding to Figure 3D***									**<0.001**
Baseline	17 (3%)	518	10 (3%)	365	5 (2%)	280	0 (0%)	39	
0-1 years	23 (3%)	759	5 (1%)	549	57 (13%)	446	8 (18%)	45	
1-2 years	21 (3%)	656	11 (2%)	518	46 (11%)	414	2 (4%)	46	
2-3 years	32 (5%)	688	8 (1%)	545	36 (8%)	466	4 (8%)	53	
3-4 years	23 (3%)	660	12 (2%)	538	43 (9%)	475	2 (3%)	58	
4-5 years	27 (4%)	652	10 (2%)	540	31 (6%)	483	2 (3%)	60	
5-6 years	26 (4%)	597	16 (3%)	524	46 (10%)	477	5 (8%)	60	
**Bloody stools – *corresponding to Figure 3E***									**<0.001**
Baseline	7 (1%)	516	4 (1%)	364	4 (1%)	279	0 (0%)	39	
0-1 years	12 (2%)	758	3 (1%)	550	15 (3%)	446	1 (2%)	45	
1-2 years	4 (1%)	658	3 (1%)	519	29 (7%)	414	3 (7%)	46	
2-3 years	5 (1%)	687	5 (1%)	543	40 (9%)	466	2 (4%)	53	
3-4 years	1 (0%)	659	2 (0%)	539	41 (9%)	478	1 (2%)	58	
4-5 years	3 (0%)	652	1 (0%)	540	40 (8%)	482	2 (3%)	60	
5-6 years	2 (0%)	597	5 (1%)	526	30 (6%)	477	2 (3%)	60	
**Bowel habits – *corresponding to Figure 3F***									**<0.001**
Baseline	12 (2%)	520	9 (2%)	363	4 (1%)	277	1 (3%)	39	
0-1 years	14 (2%)	758	13 (2%)	548	32 (7%)	445	3 (7%)	45	
1-2 years	10 (2%)	661	7 (1%)	521	31 (7%)	416	5 (11%)	45	
2-3 years	11 (2%)	693	8 (1%)	548	21 (5%)	466	2 (4%)	55	
3-4 years	18 (3%)	666	10 (2%)	538	16 (3%)	480	4 (7%)	58	
4-5 years	13 (2%)	653	12 (2%)	539	18 (4%)	488	3 (5%)	60	
5-6 years	16 (3%)	603	7 (1%)	523	16 (3%)	483	3 (5%)	60	

Abbreviations: AM=Active Monitoring, RP=Radical Prostatectomy, RT=Radiotherapy, BT=Brachytherapy, [a]P value from a likelihood ratio test comparing a mixed effects model, with and without treatment received as a covariate. Other covariates included a treatment cohort indicator (randomised/preference) and propensity scores. Baseline measures were not included, or adjusted for, based on this being an 'as treated' analysis.

***Table S5*. Adjusted symptoms from immediate radical treatments or after active monitoring corresponding to Figure S3**

Assessment period	RP		AM -> RP		P value[a]	RT		AM -> RT		P value[b]
	n (%) / *Mean (SD)*	N	n (%) / *Mean (SD)*	N		n (%) / *Mean (SD)*	N	n (%) / *Mean (SD)*	N	
**EPIC: ≥1 Pad per day**					**=0.001**					**=0.911**
Baseline	3 (1%)	362	2 (1%)	149		0 (0%)	277	1 (1%)	121	
0-1 years	197 (36%)	546	62 (48%)	129		7 (2%)	442	0 (0%)	113	
1-2 years	123 (24%)	517	25 (25%)	100		7 (2%)	414	3 (4%)	73	
2-3 years	126 (23%)	542	25 (33%)	75		9 (2%)	466	2 (4%)	53	
3-4 years	102 (19%)	535	12 (28%)	43		8 (2%)	478	1 (4%)	28	
**ICS male: Nocturia (>1 times per night)**					**=0.277**					**=0.087**
Baseline	147 (23%)	631	39 (25%)	154		104 (21%)	507	32 (25%)	129	
0-1 years	188 (27%)	685	41 (31%)	131		239 (42%)	564	61 (52%)	117	
1-2 years	126 (21%)	590	20 (20%)	101		156 (32%)	488	30 (38%)	79	
2-3 years	131 (23%)	573	14 (19%)	75		161 (32%)	500	20 (38%)	53	
3-4 years	123 (22%)	556	11 (24%)	46		162 (33%)	497	12 (41%)	29	
**EPIC: Men reporting lack of firmness**					**=0.033**					**=0.002**
Baseline	123 (35%)	352	62 (43%)	145		105 (38%)	276	56 (47%)	119	
0-1 years	514 (95%)	541	119 (96%)	124		300 (69%)	433	97 (87%)	112	
1-2 years	435 (86%)	504	89 (94%)	95		267 (66%)	402	63 (84%)	75	
2-3 years	452 (85%)	531	63 (89%)	71		308 (68%)	454	39 (74%)	53	
3-4 years	448 (84%)	534	39 (89%)	44		319 (69%)	462	21 (81%)	26	
**EPIC: Bloody stools**					**=0.478**					**=0.907**
Baseline	4 (1%)	364	1 (1%)	149		4 (1%)	279	0 (0%)	119	
0-1 years	3 (1%)	550	1 (1%)	131		15 (3%)	446	6 (5%)	115	
1-2 years	3 (1%)	519	0 (0%)	99		29 (7%)	414	6 (8%)	77	
2-3 years	5 (1%)	543	1 (1%)	72		40 (9%)	466	6 (11%)	54	
3-4 years	2 (0%)	539	2 (4%)	46		41 (9%)	478	2 (7%)	29	

Abbreviation: RP=Radical Prostatectomy, RT=Radiotherapy, AM->RP and AM->RT refer to those who commenced with active monitoring for at least one year before switching to radical prostatectomy or radiotherapy, respectively. [a]P value from a likelihood ratio test comparing a mixed effects model, with and without treatment received (RP vs. AM->RP) as a covariate. Other covariates included a treatment cohort indicator (randomised/preference) and propensity scores. Baseline measures were not included, or adjusted for, based on this being an 'as treated' analysis. [b]P value from a likelihood ratio test comparing a mixed effects model, with and without treatment received (RT vs. AM->RT) as a covariate with other covariates, described above.

Table S6. Adjusted quality of life items by treatment received corresponding to Figure S4

Assessment period	AM		RP		RT		BT		P value[a]
	n (%) / *Mean (SD)*	N	n (%) / *Mean (SD)*	N	n (%) / *Mean (SD)*	N	n (%) / *Mean (SD)*	N	
12-Item Short Form Survey (SF-12)									
**Physical weight**									**=0.012**
Baseline	*49.14 (8.32)*	794	*48.40 (7.50)*	574	*49.00 (7.68)*	448	*48.40 (8.19)*	59	
0-1 years	*49.44 (8.36)*	915	*50.26 (8.75)*	629	*50.98 (9.39)*	526	*48.28 (8.42)*	63	
1-2 years	*49.42 (8.44)*	790	*49.97 (8.52)*	555	*49.74 (8.35)*	448	*48.81 (7.65)*	57	
2-3 years	*50.21 (8.93)*	739	*50.66 (9.04)*	538	*50.98 (9.47)*	466	*49.03 (8.61)*	63	
3-4 years	*50.72 (9.24)*	660	*50.97 (9.21)*	528	*51.05 (9.45)*	461	*49.06 (9.02)*	53	
4-5 years	*50.64 (9.04)*	633	*50.93 (9.57)*	500	*52.41 (10.28)*	451	*49.36 (7.79)*	56	
5-6 years	*51.41 (9.31)*	555	*50.98 (9.63)*	471	*52.31 (9.79)*	449	*49.66 (8.51)*	57	
**Mental weight**									**=0.250**
Baseline	*46.46 (8.12)*	794	*46.29 (7.72)*	574	*45.54 (6.59)*	448	*46.53 (8.72)*	59	
0-1 years	*46.20 (7.74)*	915	*46.69 (8.89)*	629	*47.22 (8.91)*	526	*46.53 (6.53)*	63	
1-2 years	*45.77 (7.57)*	790	*46.82 (9.11)*	555	*46.52 (8.34)*	448	*45.22 (6.93)*	57	
2-3 years	*45.60 (7.68)*	739	*46.25 (8.44)*	538	*46.32 (8.18)*	466	*45.71 (8.15)*	63	
3-4 years	*45.97 (7.94)*	660	*45.75 (7.95)*	528	*46.13 (7.94)*	461	*47.11 (9.47)*	53	
4-5 years	*45.94 (7.99)*	633	*46.81 (8.67)*	500	*46.25 (8.34)*	451	*45.82 (9.27)*	56	
5-6 years	*46.16 (8.38)*	555	*46.18 (8.18)*	471	*46.63 (8.32)*	449	*46.45 (9.53)*	57	
Hospital Anxiety and Depression Scale (HADS)									
**Anxiety cases (score ≥8)**									**=0.768**
Baseline	179 (20%)	888	129 (21%)	624	93 (18%)	504	16 (27%)	59	
0-1 years	137 (14%)	995	95 (14%)	679	77 (14%)	563	6 (9%)	66	
1-2 years	109 (13%)	836	74 (13%)	586	74 (15%)	485	6 (9%)	64	
2-3 years	92 (12%)	786	74 (13%)	574	81 (16%)	502	5 (8%)	65	
3-4 years	98 (14%)	715	78 (14%)	553	61 (12%)	490	9 (16%)	58	
4-5 years	89 (13%)	674	71 (13%)	535	68 (14%)	488	4 (7%)	56	
5-6 years	65 (11%)	598	75 (14%)	518	60 (13%)	477	7 (12%)	60	
**Depression cases (score ≥8)**									**=0.027**
Baseline	54 (6%)	880	25 (4%)	622	23 (5%)	505	2 (3%)	58	
0-1 years	58 (6%)	994	54 (8%)	684	46 (8%)	561	3 (5%)	66	
1-2 years	44 (5%)	833	39 (7%)	590	34 (7%)	486	2 (3%)	64	
2-3 years	41 (5%)	793	44 (8%)	575	45 (9%)	505	4 (6%)	65	
3-4 years	48 (7%)	718	38 (7%)	552	43 (9%)	492	2 (3%)	58	
4-5 years	51 (8%)	672	42 (8%)	535	46 (9%)	491	3 (5%)	58	
5-6 years	45 (8%)	597	42 (8%)	515	37 (8%)	479	5 (8%)	60	

Abbreviation: AM=Active Monitoring, RP=Radical Prostatectomy, RT=Radiotherapy, BT=Brachytherapy, [a]P value from a likelihood ratio test comparing a mixed effects model, with and without treatment received as a covariate, adjusting for the measure at baseline. Other covariates included a treatment cohort indicator (randomised/preference) and propensity scores. Baseline measures were not included, or adjusted for, based on this being an 'as treated' analysis.

***Table S7*. Adjusted quality of life of radical treatments after active monitoring and immediate: radical treatment corresponding to Figure S5**

Assessment period	AM		AM -> RP		P value	RT		AM -> RT		P value
	n (%) / *Mean (SD)*	N	n (%) / *Mean (SD)*	N		n (%) / *Mean (SD)*	N	n (%) / *Mean (SD)*	N	
12-Item Short Form Survey (SF-12)										
**Physical weight**					**=0.554**					**=0.020**
Baseline	*48.40 (7.50)*	574	*49.18 (8.48)*	147		49.00 (7.68)	448	51.14 (9.29)	113	
0-1 years	*50.26 (8.75)*	629	*51.78 (9.51)*	129		50.98 (9.39)	526	53.37 (10.53)	110	
1-2 years	*49.97 (8.52)*	555	*49.89 (9.03)*	100		49.74 (8.35)	448	53.97 (11.03)	68	
2-3 years	*50.66 (9.04)*	538	*50.06 (9.78)*	72		50.98 (9.47)	466	52.93 (11.55)	49	
3-4 years	*50.97 (9.21)*	528	*51.26 (10.83)*	42		51.05 (9.45)	461	53.82 (11.44)	27	
**Mental weight**					**=0.224**					**=0.487**
Baseline	*46.29 (7.72)*	574	*47.40 (9.31)*	147		*45.54 (6.59)*	448	*46.33 (8.36)*	113	
0-1 years	*46.69 (8.89)*	629	*47.81 (9.75)*	129		*47.22 (8.91)*	526	*47.68 (10.03)*	110	
1-2 years	*46.82 (9.11)*	555	*47.05 (9.48)*	100		*46.52 (8.34)*	448	*47.42 (8.37)*	68	
2-3 years	*46.25 (8.44)*	538	*46.73 (9.80)*	72		*46.32 (8.18)*	466	*47.79 (9.00)*	49	
3-4 years	*45.75 (7.95)*	528	*48.28 (11.71)*	42		*46.13 (7.94)*	461	*48.01 (8.27)*	27	
Hospital Anxiety and Depression Scale (HADS)										
**Anxiety cases (score ≥8)**					**=0.630**					**=0.574**
Baseline	129 (21%)	624	33 (22%)	153		93 (18%)	504	22 (17%)	128	
0-1 years	95 (14%)	679	18 (13%)	135		77 (14%)	563	17 (15%)	117	
1-2 years	74 (13%)	586	14 (14%)	100		74 (15%)	485	10 (13%)	78	
2-3 years	74 (13%)	574	11 (15%)	75		81 (16%)	502	8 (15%)	54	
3-4 years	78 (14%)	553	7 (16%)	45		61 (12%)	490	6 (22%)	27	
**Depression cases (score ≥8)**					**=0.084**					**>0.999**
Baseline	25 (4%)	622	13 (8%)	154		23 (5%)	505	11 (9%)	128	
0-1 years	54 (8%)	684	13 (10%)	134		46 (8%)	561	12 (10%)	118	
1-2 years	39 (7%)	590	8 (8%)	101		34 (7%)	486	7 (9%)	79	
2-3 years	44 (8%)	575	9 (12%)	76		45 (9%)	505	4 (7%)	55	
3-4 years	38 (7%)	552	7 (16%)	44		43 (9%)	492	3 (11%)	27	

Abbreviation: RP=Radical Prostatectomy, RT=Radiotherapy, AM->RP and AM->RT refer to those who commenced with active monitoring for at least one year before switching to radical prostatectomy or radiotherapy, respectively. [a]P value from a likelihood ratio test comparing a mixed effects model, with and without treatment received (RP vs. AM->RP) as a covariate. Other covariates included a treatment cohort indicator (randomised/preference) and propensity scores. Baseline measures were not included, or adjusted for, based on this being an 'as treated' analysis. [b]P value from a likelihood ratio test comparing a mixed effects model, with and without treatment received (RT vs. AM->RT) as a covariate. Other covariates included the baseline measure of the symptom, a treatment cohort indicator (randomised/preference) and propensity scores.

Table S8. Adjusted interaction between treatment and age group on symptoms corresponding to Figure S4

Assessment period	AM (<65)		AM (≥65)		RP (<65)		RP (≥65)		RT (<65)		RT (≥65)		P values	
	n (%) / *Mean (SD)*	N	n (%) / *Mean (SD)*	N	n (%) / *Mean (SD)*	N	n (%) / *Mean (SD)*	N	n (%) / *Mean (SD)*	N	n (%) / *Mean (SD)*	N	Overall[a]	Pairwise[b]
**EPIC: ≥1 Pad per day**													**P=0.474**	
Baseline	0 (0%)	318	0 (0%)	202	2 (1%)	251	1 (1%)	111	0 (0%)	164	0 (0%)	113		
0-1 years	2 (0%)	459	1 (0%)	297	120 (33%)	361	77 (42%)	185	1 (0%)	266	6 (3%)	176		
1-2 years	2 (1%)	389	2 (1%)	271	71 (21%)	342	52 (30%)	175	1 (0%)	251	6 (4%)	163		
2-3 years	2 (0%)	413	1 (0%)	278	69 (20%)	353	57 (30%)	189	1 (0%)	281	8 (4%)	185		
3-4 years	2 (1%)	388	2 (1%)	267	57 (16%)	357	45 (25%)	178	3 (1%)	294	5 (3%)	184		
4-5 years	4 (1%)	392	1 (0%)	254	64 (18%)	361	45 (27%)	168	2 (1%)	299	4 (2%)	186		
5-6 years	3 (1%)	352	5 (2%)	243	59 (17%)	344	42 (26%)	161	3 (1%)	301	4 (2%)	174		
**ICS male: Nocturia (>1 times per night)**													**P=0.016**	
Baseline	82 (15%)	547	101 (29%)	347	83 (19%)	430	64 (32%)	201	56 (18%)	320	48 (26%)	187		
0-1 years	111 (18%)	611	112 (29%)	387	102 (22%)	457	86 (38%)	228	144 (41%)	349	95 (44%)	215		RP vs. AM
1-2 years	108 (21%)	505	111 (33%)	339	66 (17%)	393	60 (30%)	197	81 (27%)	303	75 (41%)	185		P=0.612
2-3 years	102 (21%)	478	119 (38%)	312	72 (19%)	378	59 (30%)	195	90 (30%)	305	71 (36%)	195		RT vs. AM
3-4 years	116 (27%)	432	108 (38%)	283	68 (18%)	369	55 (29%)	187	98 (32%)	307	64 (34%)	190		P=0.003
4-5 years	115 (28%)	413	115 (44%)	259	75 (20%)	366	46 (27%)	172	102 (34%)	297	64 (35%)	185		RP vs. RT
5-6 years	119 (33%)	357	108 (44%)	244	64 (18%)	352	50 (29%)	173	101 (33%)	302	70 (40%)	177		P=0.019
**EPIC: Men reporting lack of firmness**													**P=0.198**	
Baseline	37 (12%)	308	33 (17%)	193	35 (14%)	242	26 (23%)	111	22 (13%)	163	33 (29%)	114		
0-1 years	81 (18%)	448	62 (22%)	277	257 (72%)	356	121 (66%)	184	106 (41%)	257	80 (48%)	165		
1-2 years	64 (17%)	380	59 (23%)	258	207 (62%)	333	103 (60%)	171	80 (33%)	242	69 (45%)	154		
2-3 years	83 (21%)	398	60 (23%)	261	173 (50%)	344	101 (55%)	184	91 (34%)	268	93 (52%)	180		
3-4 years	84 (22%)	389	63 (25%)	250	180 (51%)	351	104 (57%)	181	92 (33%)	283	84 (47%)	177		
4-5 years	82 (21%)	385	70 (29%)	243	169 (47%)	360	90 (55%)	163	97 (33%)	292	89 (50%)	178		
5-6 years	82 (24%)	345	64 (28%)	227	165 (48%)	345	87 (53%)	164	101 (34%)	293	80 (48%)	166		
**EPIC: Bloody stools**													**P=0.340**	
Baseline	5 (2%)	316	2 (1%)	200	3 (1%)	251	1 (1%)	113	4 (2%)	166	0 (0%)	113		
0-1 years	8 (2%)	460	4 (1%)	298	2 (1%)	362	1 (1%)	188	10 (4%)	269	5 (3%)	177		
1-2 years	3 (1%)	385	1 (0%)	273	3 (1%)	342	0 (0%)	177	13 (5%)	251	16 (10%)	163		
2-3 years	5 (1%)	413	0 (0%)	274	5 (1%)	352	0 (0%)	191	23 (8%)	281	17 (9%)	185		
3-4 years	1 (0%)	393	0 (0%)	266	2 (1%)	356	0 (0%)	183	25 (9%)	293	16 (9%)	185		
4-5 years	3 (1%)	395	0 (0%)	257	0 (0%)	367	1 (1%)	173	23 (8%)	297	17 (9%)	185		
5-6 years	1 (0%)	355	1 (0%)	242	4 (1%)	354	1 (1%)	172	20 (7%)	301	10 (6%)	176		

Abbreviation: AM=active monitoring, RP=radical prostatectomy, RT=radiotherapy only, [a]Overall p value for the likelihood ratio test of inclusion/exclusion of an interaction term between age (continuous) and treatment group, [b]Where there was evidence of an interaction, pairwise comparisons were carried out to see where treatment effect was modified by age group

Table S9. **Adjusted symptoms and quality five years after enrolment in the CEASAR cohort or diagnosis in the ProtecT trial**

EPIC score/item	ProtecT (all)* Median (IQR) / %	CEASAR (low risk) Median (IQR) / %
**Active monitoring/surveillance (n)***	1135 (EPIC c.570)	363
**Urinary function**		
Incontinence score	100 (86, 100)	92 (73, 100)
Leakage (%)	1%	7%
Irritative score	96 (89, 100)	88 (81, 100)
Frequency of problem (%)	4%	15%
**Sexual function**		
Sexual function score	50 (29, 67)	55 (22, 85)
Erection, lack of firmness	50%	57%
**Bowel function**		
Bowel function score	96 (89, 100)	100 (92, 100)
Bloody stools	<1%	0%
**Radical prostatectomy (n)**	**Open** 750 (EPIC c.520)	**Robotic-Assisted** 675
**Urinary function**		
Incontinence score	86 (67, 94)	79 (58, 100)
Leakage (%)	6%	10%
Irritative score	96 (93, 100)	94 (88, 100)
Frequency of problem (%)	6%	12%
**Sexual function**		
Sexual function score	31 (9, 53)	48 (15, 80)
Erection, lack of firmness	71%	61%
**Bowel function**		
Bowel function score	93 (86, 96)	100 (96, 100)
Bloody stools	<1%	0%
**Radiotherapy (n)**	**EBRT[1]** 603 (EPIC c.480)	**IMRT[2]** 261
**Urinary function**		
Incontinence score	100 (86, 100)	100 (79, 100)
Leakage (%)	1%	6%
Irritative score	96 (89, 100)	94 (81, 100)
Frequency of problem (%)	4%	13%
**Sexual function**		
Sexual function score	31 (9, 53)	28 (9, 69)
Erection, lack of firmness	71%	74%
**Bowel function**		
Bowel function score	93 (86, 96)	96 (88, 100)
Bloody stools	8%	0%
**Low-dose-rate brachytherapy (n)**	77 (EPIC c. 60)	87
**Urinary function**		
Incontinence score	97 (79, 100)	100 (81, 100)
Leakage (%)	5%	5%
Irritative score	93 (88, 100)	94 (84, 100)
Frequency of problem (%)	7%	14%
**Sexual function**		
Sexual function score	42 (21, 64)	53 (24, 78)
Erection, lack of firmness	58%	61%
**Bowel function**		
Bowel function score	96 (89, 100)	100 (88, 100)
Bloody stools	3%	0%

ProtecT used EPIC-50, CEASAR used EPIC-26, [1]External Beam Radiotherapy, [2]IMRT; Intensity modulated radiation therapy low risk group patients, *ProtecT adjusted results censored at second treatment, unlike CEASAR unadjusted results in Table e5 (numbers of men unavailable for binary items). [https://jamanetwork.com/journals/jama/fullarticle/2758599]
